# Chemical heterogeneity enhances hydrogen resistance in high-strength steels

**DOI:** 10.1038/s41563-021-01050-y

**Published:** 2021-07-08

**Authors:** Binhan Sun, Wenjun Lu, Baptiste Gault, Ran Ding, Surendra Kumar Makineni, Di Wan, Chun-Hung Wu, Hao Chen, Dirk Ponge, Dierk Raabe

**Affiliations:** 1 Max-Planck-Institut für Eisenforschung, Düsseldorf, Germany; 2 Department of Mechanical and Energy Engineering, Southern University of Science and Technology, Shenzhen, China; 3 Department of Materials, Royal School of Mines, Imperial College, London, UK; 4 Key Laboratory for Advanced Materials of Ministry of Education, School of Materials Science and Engineering, Tsinghua University, Beijing, China; 5 State Key Laboratory of Hydraulic Engineering Simulation and Safety, School of Materials Science and Engineering, Tianjin University, Tianjin, China; 6 Department of Materials Engineering, Indian Institute of Science, Bangalore, India; 7 Department of Mechanical and Industrial Engineering, Norwegian University of Science and Technology, Trondheim, Norway

**Keywords:** Mechanical properties, Metals and alloys

## Abstract

The antagonism between strength and resistance to hydrogen embrittlement in metallic materials is an intrinsic obstacle to the design of lightweight yet reliable structural components operated in hydrogen-containing environments. Economical and scalable microstructural solutions to this challenge must be found. Here, we introduce a counterintuitive strategy to exploit the typically undesired chemical heterogeneity within the material’s microstructure that enables local enhancement of crack resistance and local hydrogen trapping. We use this approach in a manganese-containing high-strength steel and produce a high dispersion of manganese-rich zones within the microstructure. These solute-rich buffer regions allow for local micro-tuning of the phase stability, arresting hydrogen-induced microcracks and thus interrupting the percolation of hydrogen-assisted damage. This results in a superior hydrogen embrittlement resistance (better by a factor of two) without sacrificing the material’s strength and ductility. The strategy of exploiting chemical heterogeneities, rather than avoiding them, broadens the horizon for microstructure engineering via advanced thermomechanical processing.

## Abstract (web summary)

Typically undesired chemically heterogeneous microstructures are shown to enhance the resistance of high-strength steel against hydrogen embrittlement, with no loss in strength or ductility.

## Main

When hydrogen (H), the lightest, smallest and most abundant atom in the Universe, makes its way into a high-strength alloy (strength above ~650 MPa), the material’s load-bearing capacity is abruptly lost^1–3^. This phenomenon, known as H embrittlement, has been responsible for the catastrophic and unpredictable failure of large engineering structures in service^4,5^. Steels represent ~90% of the global metallic alloy market, which means that even small improvements in their performance have a worldwide impact. Yet high-strength steels are particularly prone to H embrittlement, as less than 1 parts per million by weight (ppmw) H is often sufficient to result in a dramatic degradation of their mechanical properties^6–9^. Such extreme sensitivity to H embrittlement, in conjunction with the often unavoidable H ingress during steel production and/or service, fuels particular concerns for the use of these materials in currently targeted carbon-free hydrogen-propelled industries and reduced-emission transportation solutions^1,10,11^.

Catastrophic failure induced by solute H is often caused by an accelerated damage evolution process involving (1) the ingress of typically only a few parts per million H, followed by its diffusion inside the microstructure, interacting with various lattice defects (for example, vacancies, dislocations and interfaces)^10,12^; (2) accelerated damage nucleation and propagation due to H-defect interactions^2,3,12–14^; and (3) the accumulation of more H around the propagating crack tips through diffusion, driven by the crack-tip stress field^2,15^, which further promotes cracking. Current engineering solutions normally include preventing H ingress by applying protective coatings^3,16^, which can fail under abrasive and corrosive environments^17^. An alternative is to design microstructures that are intrinsically resilient (for example, through grain refinement^18^ or introducing H-trapping precipitates^3,10,19^), but such measures have led to reduced strain-hardening ability and/or ductility in the H-free condition^18,19^. Microalloying in steels to form various H-trapping precipitates (for example, Ti-based and V-based carbides) can suppress internal H migration^3,20^, although it increases the materials’ cost. However, this approach loses effectiveness when all H traps are filled to saturation^21,22^, which can readily occur due to the typically low volume fraction of these precipitates (below 1% (ref. ^23^)), that is, the limited H storage volume. Developing inexpensive and scalable microstructure solutions that enable both an intrinsically high resistance to H and high mechanical performance thus still remains a fundamental challenge.

**Fig. 1:**
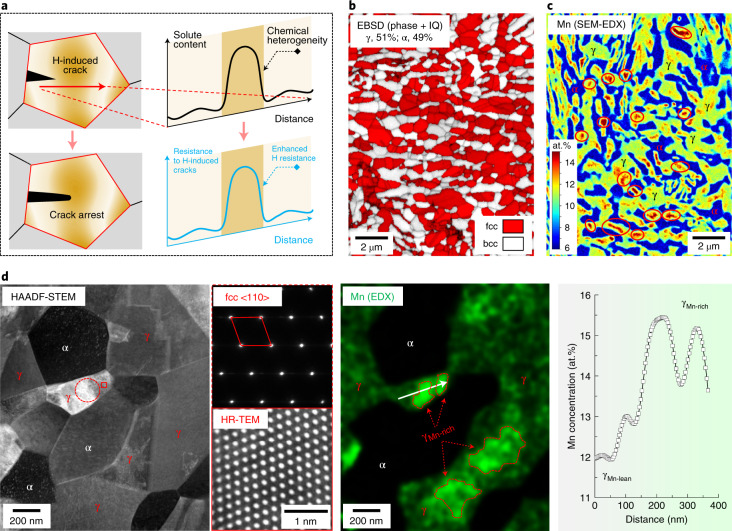
Concept of chemical-heterogeneity-induced crack arresting as a measure against H embrittlement and the microstructure of a high-strength steel designed with a heterogeneous Mn distribution inside austenite. **a**, Schematic image of the concept, showing a H-induced crack propagation crossing a designed solute-rich buffer region, with the solute concentration profile and the corresponding crack resistance schematically shown on the right side. **b**, Electron backscatter diffraction (EBSD) phase plus image quality (IQ) map showing the austenite–ferrite dual-phase microstructure. fcc, face-centred cubic; bcc, body-centred cubic. **c**, Scanning electron microscopy (SEM)-based energy-dispersive X-ray spectroscopy (EDX) map revealing the overall Mn distribution pattern in the microstructure. The chemical buffer zones are those regions where Mn is highly enriched (~14–16 at.% Mn) inside the austenite phase (some of them are marked by elliptical frames). **d**, High-angle annular dark-field scanning transmission electron microscopy (HAADF-STEM) observation with EDX analysis, showing the existence of multiple Mn-rich zones inside one austenite crystal cluster or even one austenite grain. The selected area electron diffraction and high-resolution transmission electron microscopy (HR-TEM) images taken, respectively, from the marked circular and rectangular frames are placed on the right side of the STEM image. The EDX line profile is taken from the area marked by a white arrow in the EDX map.

Here, we provide a solution based on a strategy of exploiting solute heterogeneity in steels’ microstructure constituents. The well-designed local variations in composition serve to enhance crack resistance locally, creating buffer zones that arrest H-induced microcracks that otherwise would rapidly propagate inside or along H-attacked phases or interfaces (Fig. 1a). We demonstrate our approach on a lightweight high-strength medium-manganese (Mn) steel (0.2C–10Mn–3Al–1Si in weight per cent). Assisted by Computer Coupling of Phase Diagrams and Thermochemistry (CALPHAD), a well-adjusted design of the Mn heterogeneity inside the austenite phase produces a high number density of microscopically confined Mn-rich buffer regions dispersed throughout the sample. During deformation of the alloy, the dynamic transformation from soft austenite to hard martensite is locally suppressed inside these buffer regions by the increased mechanical stability associated with their locally higher Mn content. As a result, the microstructure evolves into a high dispersion of softer islands embedded in the hard matrix, which frequently renders H-induced microcracks to be blunted and arrested. The chemical heterogeneity is here realized through a heat treatment with incomplete Mn partitioning/homogenization. The processing can be fully guided by the CALPHAD method and is readily scalable to established and affordable industrial processing routes. Also, it can be generalized to other types of high-strength steels with the same aim, namely, to lend their microstructures enhanced resistance against H embrittlement.

Our high-strength steel (strength level ~1 GPa) contains submicrometre-sized domains of ferrite (α) and austenite (γ, Fig. 1b) and shows the transformation-induced plasticity (TRIP) effect, characterized by the deformation-driven displacive transformation from face-centred cubic austenite into body-centred tetragonal α′-martensite (the crystal structure of the latter phase is similar to ferrite). The TRIP effect is a particularly efficient strain-hardening mechanism for metallic materials, often leading to a leap forward in their mechanical properties^24^. It drives the development of most modern, advanced high-strength steels^24,25^ and some maraging^26^ and stainless^25,27^ steels. However, its phase transformation product (martensite) and the associated hetero-interfaces are strongly prone to H-induced cracking, which is the major reason leading to the premature failure of such materials when exposed to H (refs. ^15,19^). To manipulate the chemical heterogeneity inside the austenite phase, we designed a multi-step annealing process compatible with current industrial practice (Methods). The process uses the slow kinetics of Mn homogenization between different Mn-containing austenite regions rapidly formed during a series of carefully designed annealing stages (Supplementary Fig. 1 and Supplementary Note 1). A compositional variation of ~5 at.% Mn is produced inside austenite (Fig. 1c,d), which consists of Mn-rich stable buffer islands (γ_Mn-rich_ containing ~14–16 at.% Mn) and the surrounding Mn-lean metastable austenite regions (γ_Mn-lean_ containing ~11–12 at.% Mn). Multiple such Mn-rich zones are formed in single or multiple contiguous austenite grains (Fig. 1d). Taking advantage of the alternately arranged dual-phase microstructure (Fig. 1b,c) and its ultrafine grain size (~0.6 μm), we achieved a high number density of γ_Mn-rich_ (above ~2 × 10^18^ m^–3^) dispersed throughout the material. Its total volume fraction, however, remains below 5%, due to its small size (~0.05–0.5 μm).

**Fig. 2:**
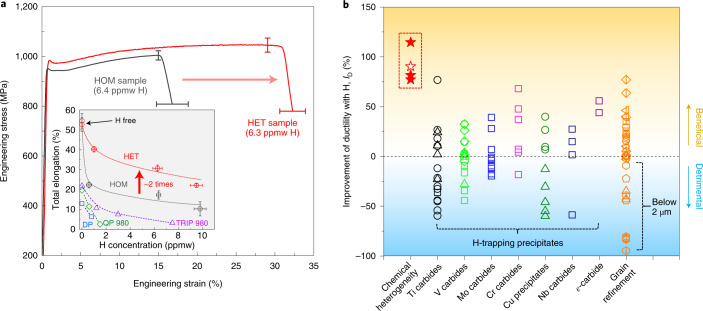
Improvement of the resistance to H embrittlement via designing chemical heterogeneity. **a**, Slow strain rate tensile properties of the HET sample in comparison to the HOM reference material. Both samples were subjected to the same H-precharging conditions (similar H amount). Representative tensile curves of samples with a total H concentration of ~6.5 ppmw are shown here. The inset shows the ductility of the two samples as a function of the total H concentration. The literature data of testing on commercial high-strength steels (TRIP 980^8^, QP 980^9^ and DP^7^ steels) with a similar strength level (that is, yield strength above ~600 MPa and tensile strength of ~1,000 MPa) are also included, indicating the required resistance to H embrittlement in automotive applications. The error bars represent standard deviations from repeated tests. **b**, Comparison between our approach and other H-resistance-enhancing methods reported in the literature, in terms of their effects on the improvement of the tensile ductility in the presence of H (*I*_D_ = [(*ε*_f-H,after_ *–* *ε*_f-H,before_)/*ε*_f-H,before_ ] × 100%, where *ε*_f-H,before_ and *ε*_f-H,after_ are the total elongations or fracture strains of the H-containing specimens before and after applying the resistance-enhancing approach, respectively). Our tests performed on both H-precharged specimens (solid red star symbols in red dashed box) and specimens under continuous electrochemical charging during deformation (hollow star symbol; results also shown in Supplementary Fig. 11) are shown. The literature data are from different materials including low-alloy ferritic/martensitic steels, pipeline steels, various advanced high-strength steels, maraging steels, various austenitic stainless steels and high-entropy alloys, subjected to electrochemical or gaseous H_2_ precharging or in-situ H charging. More details about the symbols in this figure, the values and the references are shown in Supplementary Fig. 5 and Supplementary Table 2.

Our approach of designing chemical heterogeneity results in a notably improved resistance to H embrittlement. This is revealed by comparing our steel having a heterogeneous Mn distribution (referred to as the HET sample) to a reference specimen that has the same bulk composition and a similar microstructure but has been processed to obtain a nearly homogeneous Mn distribution inside austenite (HOM sample, as shown in Supplementary Fig. 2 and Supplementary Table 1). Both samples were subjected to the same H-precharging condition, which yields a similar total H concentration (~1–10 ppmw controlled by varying the electrochemical charging conditions) and similar H penetration depth in the two samples, as suggested by thermal desorption spectroscopy results (Supplementary Fig. 3) and permeation testing results (Methods), respectively. At the same H concentration, the HET sample shows nearly twice the total elongation (~22.1–40.3%) compared with the HOM sample (~10.3–22.2%), determined by slow strain rate (~8 × 10^–5^ s^–1^) tensile testing (Fig. 2a). The mechanical performance under the H-free condition, however, is nearly the same for the two samples (Fig. 2a and Supplementary Fig. 4). The strain-hardening ability, controlled by the overall kinetics of the deformation-induced austenite-to-martensite transformation (the TRIP effect), is not affected by the presence of the small fraction of γ_Mn-rich_ (below 5 vol%), as demonstrated in Supplementary Fig. 4. This reveals the ability of the manipulated chemical heterogeneity to reconcile the most important, yet often mutually exclusive, material’s features in this field, namely, high H resistance and high strain hardening. The latter is particularly important for maintaining ductility (and formability) in high-strength alloys, as it counteracts Considère-type plastic instability.

We further compare our microstructure engineering approach with other previously reported methods for enhancing the resistance to H embrittlement (Fig. 2b and Supplementary Fig. 5). The H resistance is here characterized by both the tensile ductility in the presence of H and by the so-called H embrittlement index (the ratio of ductility among the samples tested with and without H (ref. ^28^)). While the introduction of H-trapping precipitates, generally adopted in ferritic/martensitic steels^10,19,20,22^, can improve the H resistance before H saturation, the improvement is typically below ~75% (Fig. 2b and Supplementary Fig. 5) and is bounded by the limited H storage volume. The formation of precipitates, even though confined to the nanoscale, can also result in detrimental effects, for instance, due to the introduced elasto-plastic deformation fields inside the adjacent matrix that increase the uptake of diffusible H (refs. ^3,22^). Grain refinement improves the H embrittlement index generally in austenitic materials (Supplementary Fig. 5). However, the ductility in fine-grained materials (especially with a grain size below 2 μm) can be seriously degraded in both the H-containing and H-free conditions (Fig. 2b and Supplementary Table 2). This is due to the limited strain hardening of such materials, which can promote early plastic instability associated with H-induced local plasticity (that is, softening)^29^. Our approach does not suffer from these limitations and achieves a higher improvement in H-embrittlement resistance (up to 122%), surpassing the effects achieved by the other two widely adopted strategies.

**Fig. 3:**
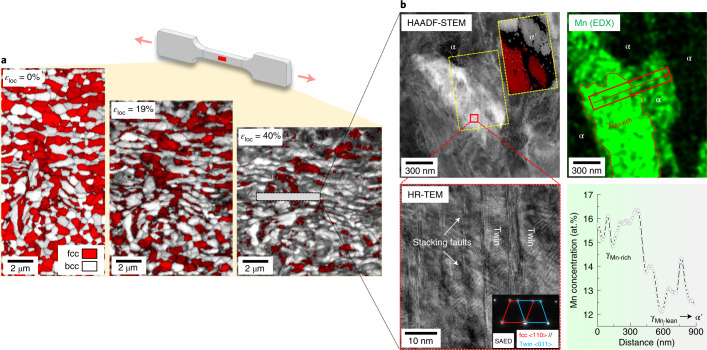
Microstructure evolution of the HET sample under the H-free condition. **a**, The schematic illustrates the tensile specimen subjected to deformation and ex-situ EBSD probing, with the red spot showing where the EBSD images were taken. Ex-situ electron backscatter diffraction observation up to a local tensile strain (*ε*_loc_) of 40%, showing the partial austenite-to-martensite transformation. **b**, STEM and EDX analyses generated roughly from the subsurface of the rectangular frame marked in **a**. The inset in the HAADF-STEM image is a transmission Kikuchi diffraction phase plus IQ map, taken from the yellow rectangular frame. The EDX line profile is taken from the area marked by an arrow in the EDX map. The inset in the HR-TEM image shows the selected area electron diffraction (SAED) of the twinned structure, with diffraction spots for the fcc matrix and the fcc twin, marked by two quadrilaterals in different colours.

**Fig. 4:**
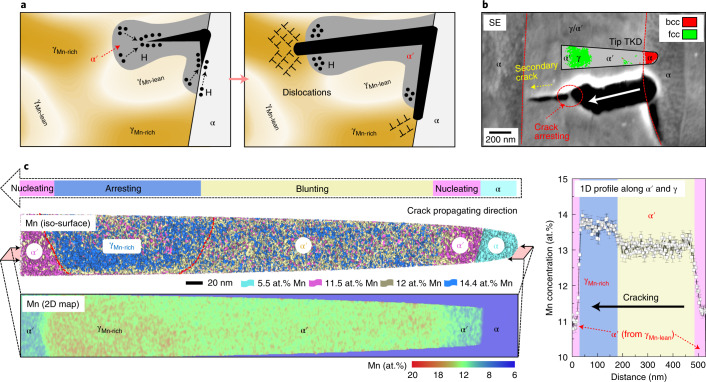
Chemical-heterogeneity-induced arresting of H-induced cracks. **a**, Schematic illustration showing the crack-arresting mechanisms. The stable γ_Mn-rich_ resists deformation-induced phase transformation, which allows it to blunt and arrest intruding H-induced microcracks owing to the enhanced plastic compliance and reduced H diffusivity therein. **b**, A representative blunted and arrested H-induced crack in the H-charged and fractured chemical-heterogeneity-manipulated steel (total H amount 6.3 ppmw). The crack was probed by secondary electron (SE) imaging from the area close to the fracture surface and specimen side edge, where H is nearly saturated. The inset is the transmission Kikuchi diffraction (TKD) phase map for the APT tip, which is placed at the region exactly where the tip was lifted out. The red dashed lines mark the interface between ferrite (α) and austenite/martensite (γ/α´). The white arrow indicates the crack propagation direction. **c**, The APT results for the tip shown in **b**, containing an iso-composition surface map for Mn, a two-dimensional (2D) Mn concentration map and a one-dimensional (1D) Mn profile across α′ and γ (the error bars represent standard deviations). Colours (pink, yellow, blue) indicate different Mn contents.

Next we validate the local resistance provided by the Mn-rich buffer zones (γ_Mn-rich_) to H-induced microcracks and unveil the underlying crack-arresting mechanisms. First we focus on the deformation behaviour of the austenite within the Mn-rich regions (Fig. 3). The higher Mn content increases the austenite’s mechanical stability locally by reducing the chemical driving force for the austenite-to-martensite phase transformation. Therefore, unlike the austenite in other regions where α′-martensite forms upon straining through the TRIP effect, the Mn-rich austenite zones resist phase transformation (Fig. 3a,b) and instead deform through the gliding of partial dislocations and formation of nano-twins (high-resolution transmission electron microscopy and selected area electron diffraction results in Fig. 3b) due to the increased stacking fault energy^30^. The capacity of these γ_Mn-rich_ domains to maintain their phase stability turns them into plastically compliant buffer zones that can arrest H-induced cracks that intrude from the neighbouring transformed regions. The arresting mechanism, as illustrated schematically in Fig. 4a, works through two effects: (1) austenite has higher H solubility and lower H diffusivity than α′-martensite (both differing by more than two orders of magnitude^6,31^), and it thus serves as a H-trapping zone, slowing down H migration^3,6,15^; and (2) the enhanced plastic compliance and flow of austenite at the crack tips result in crack blunting. The blunt cracks are frequently observed in the current H-charged and fractured HET sample and are often surrounded by untransformed austenite (Supplementary Fig. 6). In these austenite regions, electron backscatter diffraction analysis shows an increase in the point-to-point misorientation angle close to the H-induced cracks (Supplementary Fig. 6), suggesting a strong plastic deformation at the propagating crack tips^32^. Such a crack-blunting mechanism marks a distinct advantage of the current approach, as it operates even when all the traps are saturated by H, for instance after prolonged exposure to a H-abundant environment.

We used atom probe tomography (APT) to analyse the nanoscale composition in the region adjacent to a typical arrested H-induced crack in our heterogeneity-manipulated steel (Fig. 4b,c). The H-induced crack preferably nucleates at the interface between ferrite and α′-martensite (transformed from γ_Mn-lean_ containing ~11 at.% Mn). Detailed APT data analysis of such a hetero-interface reveals a tendency for H segregation (Supplementary Fig. 7 and Supplementary Note 2), which supports the role of H in promoting interface decohesion^33,34^. The nucleated crack is, however, arrested within the Mn-rich zone (~14 at.% Mn), where austenite resists load-driven phase transformation (Fig. 4b,c). Further crack propagation is thus no longer possible, and, upon further deformation, new cracks would nucleate in other Mn-lean regions, a process with a high energy barrier^35^. In some cases (~21% of the probed H-induced microcracks; Supplementary Fig. 8), the Mn-rich zones are observed to remain as ligaments that are capable of bridging the crack wake (as shown in Fig. 4b). This crack bridging effect will further reduce the crack-tip stress intensity and continuously suppress or respectively delay crack propagation^35^.

The lattice structure change associated with the deformation-induced phase transformation brings the H atoms from their initial low-mobility solute state in the host austenite to a super-saturated and highly mobile state inside the product martensite^6,36,37^. This phase-transformation-driven H release promotes damage evolution inside of the freshly formed martensite or along the associated hetero-interfaces^15^. In homogeneous TRIP-aided materials (for example, the HOM sample), H-induced cracks propagate in an autocatalytic (or accelerated) process, as the high stress and strain concentrations near the nucleated crack tip trigger more α′-martensite formation (as supported by Supplementary Fig. 9), thus liberating more mobile H. The crack-arresting effect created by the dispersed Mn-rich buffer zones thus frequently interrupts this damage evolution chain, leading to superior H-embrittlement resistance (Fig. 2). This is supported by the statistical crack analysis conducted on H-charged and fractured samples, which shows that the length of H-induced cracks near the fracture surface, regardless of their types (intergranular or transgranular), remains much shorter in the HET sample compared to the HOM counterpart (as quantified in Supplementary Fig. 10).

To further validate the enhancing effects of our approach on H resistance, we also performed tests directly under H exposure, by in-situ electrochemical charging and in-situ H-plasma charging (Methods). In both types of H-containing environments, our approach demonstrates a greatly improved fracture resistance, as characterized by the almost doubled ductility (~11.8 ± 1.3% for the HET steel versus ~6.2 ± 0.6% for the HOM sample; Supplementary Fig. 11) under continuous electrochemical charging and by the greatly reduced (by a factor of two) area of brittle regions in the fracture surface of specimens loaded under H-plasma charging (Supplementary Fig. 12). These results suggest that the presence of a high density of Mn-rich buffer zones not only suppresses internal H migration and internal crack propagation, but also mitigates in-service H environment embrittlement. The mechanism thus shows general effectiveness in enhancing a material’s tolerance against intruding H, no matter how and when H exposure occurs or what the specific operating H-embrittlement mechanism is (Supplementary Fig. 12 and Supplementary Note 3); its industrial relevance is further discussed in Supplementary Note 4.

The microstructure introduced here has been mainly designed to prove the proposed concept of chemical-heterogeneity-induced crack arresting. More efficient crack blocking is likely to be achieved by further manipulating the number density and size of the γ_Mn-rich_ regions via controlling thermomechanical processing parameters. The principle, based on a Griffith-type energy balance consideration, lies in the rationale that a larger Mn-rich buffer zone essentially requires a higher plastic energy for crack growth or opening, and a higher number density of γ_Mn-rich_ reduces the initial size of H-induced microcracks, which decreases the stress field at the crack tip.

To conclude, we turned chemical heterogeneity, normally undesired owing to its detrimental effect on materials’ conventional damage tolerance, into a mechanism that enhances the intrinsic resistance to H embrittlement. To avoid heterogeneities, alloys are usually subjected to costly high-temperature and lengthy homogenization treatments that have a large negative environmental footprint. Our approach is counterintuitive: we designed and exploited chemical heterogeneity, rather than avoiding it, to arrest H-induced microcracks and suppress their propagation. The underlying thermodynamic principle, used here for developing microstructures with a specific degree of chemical heterogeneity, lies in the high kinetic mismatch between phase transformation and solute diffusion, an effect generally applicable to alloyed steels^38,39^ (and some Ti alloys^40^). Our approach can hence be transferred to a large variety of high-performance steels containing metastable austenite, targeting different application fields (for example, various TRIP-aided advanced high-strength steels, metastable stainless steels and maraging-TRIP steels), using the crack-resistance-enhancing mechanisms introduced in this work. Furthermore, our strategy of utilizing chemical heterogeneity is expected to provide important insights into other advanced metal processing techniques such as powder metallurgy and additive manufacturing, where multiple options might exist to manipulate solute distribution or patterning^41–43^. In that context, the unique composite effect caused by solute heterogeneity—that is, the combination of high crack resistance provided by local chemical fluctuations and high mechanical performance arising from other microstructure ingredients—can also be extended to other alloys in which a compositional dependence of H resistance exists.

## Methods

### Alloy fabrication

The chemical composition of the material employed in this work is 0.2C–10.2Mn–2.8Al–1Si in weight per cent (0.8C–9.9Mn–5.5Al–1.9Si in atomic per cent). Its mass density was calculated to be ~7.5 g cm^–^^3^ based on equations in ref. ^44^, which is ~4% lighter than iron and ~6% lighter than common austenitic stainless steels. Casting was performed in a vacuum induction furnace. The steel ingots were then reheated to 1,230 °C and hot rolled to ~3 mm above 750 °C, followed by air cooling to room temperature. The hot-rolled plates consist of a predominately martensitic structure. In order to produce a heterogeneous Mn distribution inside the austenite, three processing steps were applied after hot rolling, including (1) a first intermediate annealing conducted at a low intercritical temperature (here 700 °C for 2 h) just above the Ac1 temperature, the lowest temperature at which austenite can form on heating at a specified heating rate (674 °C determined by dilatometry); (2) a second step of a cold-rolling process with a thickness reduction of ~50%; and (3) the final intercritical annealing at 750 °C for 5 min. The role of the different processing steps for the microstructural evolution is described in Supplementary Note 1. Parameter selection for the final annealing treatment was guided by a diffusion-controlled transformation (the DICTRA module) simulation, using the TCFE7 and MOB2 databases. The simulation results using the selected final annealing parameters (750 °C for 5 min) are shown in Supplementary Fig. 1. Note that steps 2 and 3 are essentially the same as the established processing routes used for this type of alloy, and step 1 can be readily implemented into current industrial practice (for example, by combining it with the coiling process). The reference HOM sample was directly cold rolled to the same thickness reduction without the intermediate annealing step, followed by the final intercritical annealing at the same temperature (750 °C). The specimen treated by this ‘conventional’ processing route shows a relatively homogeneous Mn distribution inside austenite (Supplementary Fig. 2). Other microstructural characteristics including phase constituents, fraction and grain size are very similar for the HET and HOM samples (Supplementary Table 1).

### Hydrogen charging and mechanical testing

The susceptibility to internal H embrittlement of the HET and HOM samples was evaluated by H precharging and slow strain rate tensile tests conducted at an initial strain rate of ~8 × 10^–5^ s^–1^. Tensile experiments were carried out using a Kammrath and Weiss stage equipped with the digital image correlation technique. Tensile specimens with a gauge length of 4 mm and thickness of ~1.1 mm were used. Before tensile testing, electrochemical H charging was performed on tensile specimens in an aqueous solution containing 3 wt% NaCl and 0.3 wt% NH_4_SCN at room temperature. A platinum foil was used as the counterelectrode. The total H concentration was controlled by varying the charging time (~0.5–48 h) and current density (~5–15 A m^–2^). The time interval between the end of H precharging and the start of tensile testing was less than 15 min. The H loss during this short time is negligible.

To evaluate the materials’ susceptibility to H environment embrittlement, we performed slow strain rate tensile tests under in-situ H charging to enable a continuous H ingress. Two types of in-situ charging methods with different H fugacities were used. One is electrochemical H charging using the same electrolyte as that for H precharging and a current density of 5 A m^–2^. The tests under this charging method were performed at an initial strain rate of 1 × 10^–5^ s^–1^ using a hydraulic testing machine (Zwick/Roell). The detailed experimental set-up is described elsewhere^45^. Specimens with a gauge length of 15 mm and width of 3 mm were used. The other charging method is H-plasma charging, which was performed in a Quanta 650 FEG environmental scanning electron microscope (ThermoFisher). Single-edge notched specimens (gauge length 10 mm and width 4 mm; 90° V-notch with a depth of 0.5 mm) were loaded using a Kammrath and Weiss tensile module inside the environmental scanning electron microscope (initial global strain rate 10^–4^ s^–1^), during which the plasma phase was ignited and continuously injected by an Evactron Model 25 Zephyr plasma source (XEI Scientific) using pure H gas as the process gas. More details about the H-plasma charging can be found elsewhere^46,47^. Although the H influence on the macroscopic tensile properties is difficult to detect with the current H-plasma charging set-up (partial pressure of the plasma phase is only around 40 Pa; Supplementary Note 3), this charging method provides a distinct contamination-free condition that is particularly useful for revealing detailed features of fracture surfaces.

### Thermal desorption spectroscopy and permeation testing

Thermal desorption spectroscopy experiments were performed using a Hiden TPD Workstation to measure the H concentration in precharged specimens. Specimens with a dimension of 10 × 15 × 1.1 mm^3^ were used, and were subjected to the same H-precharging condition as that for tensile specimens. The tests were started within 15 min after H charging. The total H concentration was determined by measuring the cumulative desorbed H from room temperature to 800 °C. The H diffusivity in the investigated samples was studied by permeation tests using a highly sensitive scanning Kelvin probe method^48^. Membranes with a thickness of ~80–90 μm were prepared from the steel sheets to be probed, which were then coated with an ~100 nm Pd layer by physical vapour deposition (Leybold Univex 450) on the detection side. The H permeation through the specimen immediately enters into the Pd layer due to the lower chemical potential of H in Pd than in steel. The increase of the H concentration in the Pd layer then leads to a reduction in the surface potential that can be very sensitively measured by the Kelvin probe method^48^. The permeation curves obtained by this method can be used for calculating the effective diffusion coefficient of H, which is directly comparable with the traditional Devanathan–Stachurski method^48,49^. The effective diffusion coefficient, determined from the breakthrough time^50^, is 6.0 (±1.7) × 10^–14^ m^2^ s^–1^ for the non-deformed HOM specimen and 8.3 (±1.2) × 10^–14^ m^2^ s^–1^ for the non-deformed HET specimen. The results reveal a similar H diffusivity in the two specimens, or even slightly faster H diffusivity in the HET sample due to its slightly larger grain size (lower interface density, that is 4,533 mm mm^–2^ for the HET sample versus 5,013 mm mm^–2^ for the HOM sample, measured from electron backscatter diffraction). Therefore, a similar penetration depth of H would be achieved for the two samples (or even slightly deeper for the HET sample) at the same H-precharging condition.

### Microstructure characterization

The SEM observation was conducted using a Zeiss Sigma 500, a Zeiss-Merlin and a JEOL JSM-6500F field emission SEM instrument (Zeiss Sigma 500 and Zeiss-Merlin for secondary electron imaging; Zeiss Sigma 500 and JEOL JSM-6500F for electron backscatter diffraction). Electron backscatter diffraction data were analysed using the TSL-OIM software package. SEM-based EDX was used to provide an overview of Mn distribution. It was conducted in a Hitachi SU-8230 cold-emission SEM instrument, which was operated at an accelerating voltage of 10 kV using a Bruker Flat Quad 5060F annular silicon drift detector. This high-collection-efficiency detector^51^ was used to provide high signal-to-noise-ratio X-ray images to improve the detection limit for Mn and other elements of the alloy. The weight fraction values in EDX mapping were further computed using the f-ratio method, which was calibrated based on Monte Carlo modelling results^52,53^. The number density of γ_Mn-rich_ per unit area was measured based on the SEM-based EDX mapping, and it was then converted to the value per unit volume using the relation in ref. ^54^ and the measured maximum size of γ_Mn-rich_ (~0.5 μm); that is, the value calculated is the lower bound. STEM imaging and STEM–EDX were performed in a probe aberration-corrected TEM/STEM instrument (FEI Titan Themis 60-300) with an acceleration voltage of 300 kV. For HAADF imaging, a probe semiconvergence angle of 17 mrad and inner and outer semicollection angles ranging from 73 to 200 mrad were used. High-resolution imaging and selected area electron diffraction were carried out in an image aberration-corrected TEM instrument (FEI Titan Themis 80-300) operated at 300 kV. Thin foils for STEM and transmission Kikuchi diffraction were prepared by a site-specific lift-out procedure^55^ using a dual-beam focused ion beam instrument (FEI Helios Nanolab 600i). Transmission Kikuchi diffraction analysis was conducted using a FEI Scios focused ion beam microscope at 20 kV. APT was carried out using a LEAP 5000X HR instrument (Cameca Instruments) equipped with a reflection lens. Site-specific APT tips were prepared using a dual-beam SEM/focused-ion-beam instrument (FEI Helios Nanolab 600i) via an in-situ lift-out procedure^56^. Before APT measurements, transmission Kikuchi diffraction was performed on the polished APT tips inside the SEM instrument (FEI Helios Nanolab 600i). APT experiments were operated in laser pulse mode with a pulse rate of 200 kHz, pulse energy of 45 pJ, specimen temperature of 60 K and detection rate of 10 ions per 1,000 pulses. The analysis of the APT data was performed by IVAS 3.8.4 software.

## Online content

Any methods, additional references, Nature Research reporting summaries, source data, extended data, supplementary information, acknowledgements, peer review information; details of author contributions and competing interests; and statements of data and code availability are available at 10.1038/s41563-021-01050-y.

## Supplementary information

Supplementary Information Supplementary Figs. 1–12, Tables 1 and 2, Notes 1–4 and references.

## Data Availability

The data presented in Fig. 2b are available in Supplementary Table 2. The data for other plots within this work are available from the corresponding authors on request.
