# Supplementary material for: Chemical heterogeneity enhances hydrogen resistance in high-strength steels
Source: Nat Mater. 2021 Jul 8;20(12):1629–34. doi: 10.1038/s41563-021-01050-y (PMC8610813; doi:10.1038/s41563-021-01050-y)
Supplement: Supplementary file 1 — Supplementary Figs. 1–12, Tables 1 and 2, Notes 1–4 and references. [file 41563_2021_1050_MOESM1_ESM.pdf]

nature portfolio

https://doi.org/10.1038/s41563-021-01050-y

Supplementary information

Chemical heterogeneity enhances hydrogen resistance in high-strength steels

In the format provided by the authors and unedited

Supplementary information

Chemical heterogeneity enhances hydrogen resistance in high-strength steels

Binhan Sun[1*], Wenjun Lu[1,2], Baptiste Gault[1,3], Ran Ding[4,5], Surendra Kumar Makineni[1,6], Di Wan[7], Chun-Hung Wu[1], Hao Chen[4], Dirk Ponge[1] and Dierk Raabe[1*]

[1] Max-Planck-Institut für Eisenforschung GmbH, Max-Planck-Straße 1, 40237 Düsseldorf, Germany.
[2] Department of Mechanical and Energy Engineering, Southern University of Science and Technology, 518055 Shenzhen, China.
[3] Department of Materials, Royal School of Mines, Imperial College, Exhibition Road, SW7 2AZ London, UK.
[4] Key Laboratory for Advanced Materials of Ministry of Education, School of Materials Science and Engineering, Tsinghua University, 100084 Beijing, China.
[5] State Key Laboratory of Hydraulic Engineering Simulation and Safety, School of Materials Science and Engineering, Tianjin University, 300354 Tianjin, China.
[6] Department of Materials Engineering, Indian Institute of Science, 560012 Bangalore, India.
[7] Department of Mechanical and Industrial Engineering, Norwegian University of Science and Technology, Richard Birkelands vei 2B, 7491 Trondheim, Norway.
*Correspondence to: b.sun@mpie.de (B.S.); d.raabe@mpie.de (D.R.).

Table of Contents:

Fig. S1 | As-cold rolled microstructure of the HET sample and DICTRA simulation for the final annealing process 2
Fig. S2 | Microstructure of the HOM sample before and after annealing 3
Table S1 | Detailed microstructural information of the HET and HOM samples 4
Fig. S3 | Representative TDS results of the H pre-charged HET and HOM specimens 5
Fig. S4 | Tensile property and deformation-induced phase transformation under H-free condition 6
Fig. S5 | Comparison to other H-resistance enhancing methods in term of I_{HEI} 7
Table S2 | Details of the data presented in Fig. 2b and Supplementary Fig. 5 8
Fig. S6 | Typical H-induced cracks in the HET sample pre-charged with ~6.5 wt ppm H 12
Fig. S7 | H segregation at the α-α' interface 13
Fig. S8 | Proportions of H-induced microcracks with and without bridging ligaments in the HET sample 14
Fig. S9 | Typical H-induced cracks in the HOM sample pre-charged with ~6.5 wt ppm H 15
Fig. S10 | Statistical analysis of H-induced cracks in the HET and HOM samples 16
Fig. S11 | Tensile testing results for samples under *in-situ* electrochemical H charging 17
Fig. S12 | Fracture surface analysis of samples deformed under vacuum inside the SEM chamber and under H atmosphere (*in-situ* H-plasma charging) 18
Supplementary Note
1. Role of different processing steps for the microstructure evolution 20
2. H segregation at the interface between ferrite and α'-martensite 20
3. Difference between H pre-charging and *in-situ* H charging in terms of H ingress, distribution and H embrittlement mechanisms 21
4. Industrial relevance of this work 22
Supplementary References 25

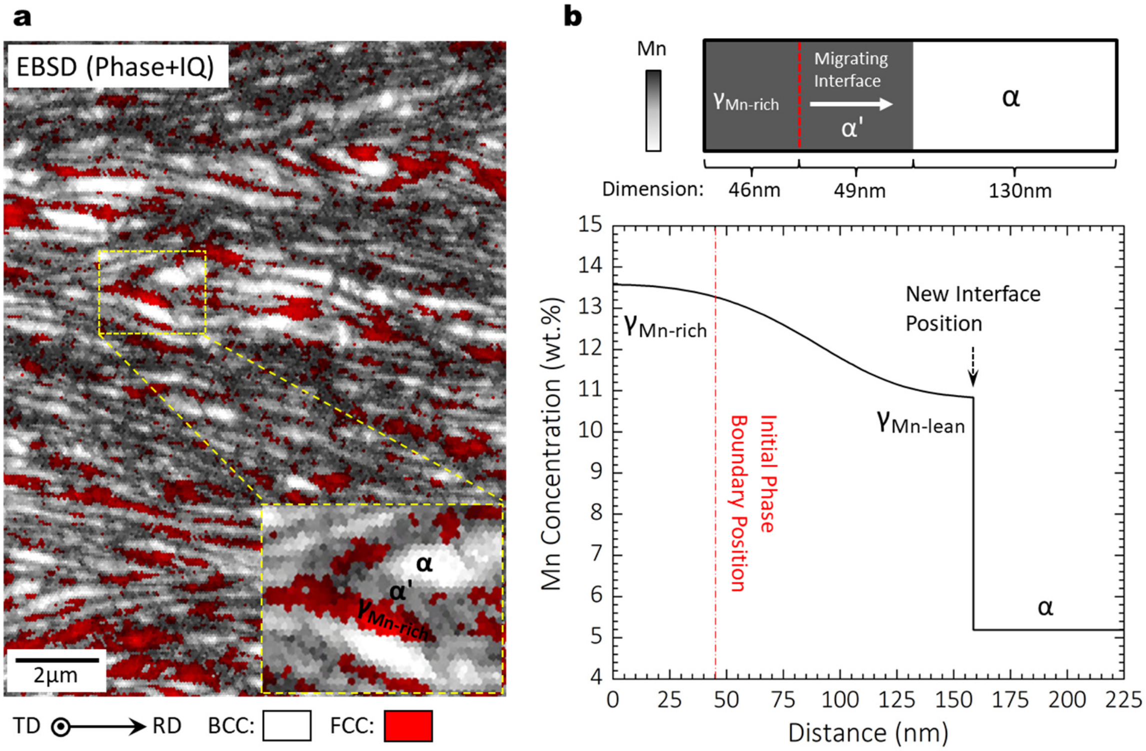

Supplementary Fig. 1 | a, Electron backscatter diffraction (EBSD) phase plus image quality (IQ) map showing the as-cold rolled microstructure of the chemical heterogeneity-manipulated (HET) sample. It consists of ferrite and Mn-rich austenite formed during the first intermediate annealing, and the latter phase partially transforms to α'-martensite during the cold rolling process. **b, DICTRA simulation results of austenite reversion in the HET sample during the final intercritical annealing step at 750 ˚C for 5 min.** The sketch of the simulation setup is also added, which was determined based on the probed cold rolled microstructure (shown in **a**). The local equilibrium condition was assumed at the moving interface. Since the initial stage of austenite reversion (controlled by rapid C diffusion and then Mn diffusion inside ferrite [1,2]) is much faster than Mn diffusion/homogenization inside austenite [1,2], sufficient reverted $\gamma_{Mn\text{-}lean}$ can form before a substantial Mn redistribution occurs between $\gamma_{Mn\text{-}lean}$ and $\gamma_{Mn\text{-}rich}$. It is demonstrated that compared with the pre-existed $\gamma_{Mn\text{-}rich}$, the newly reverted austenite ($\gamma_{Mn\text{-}lean}$) contains a lower Mn content. A heterogeneous Mn distribution inside the austenite phase is thus achieved.

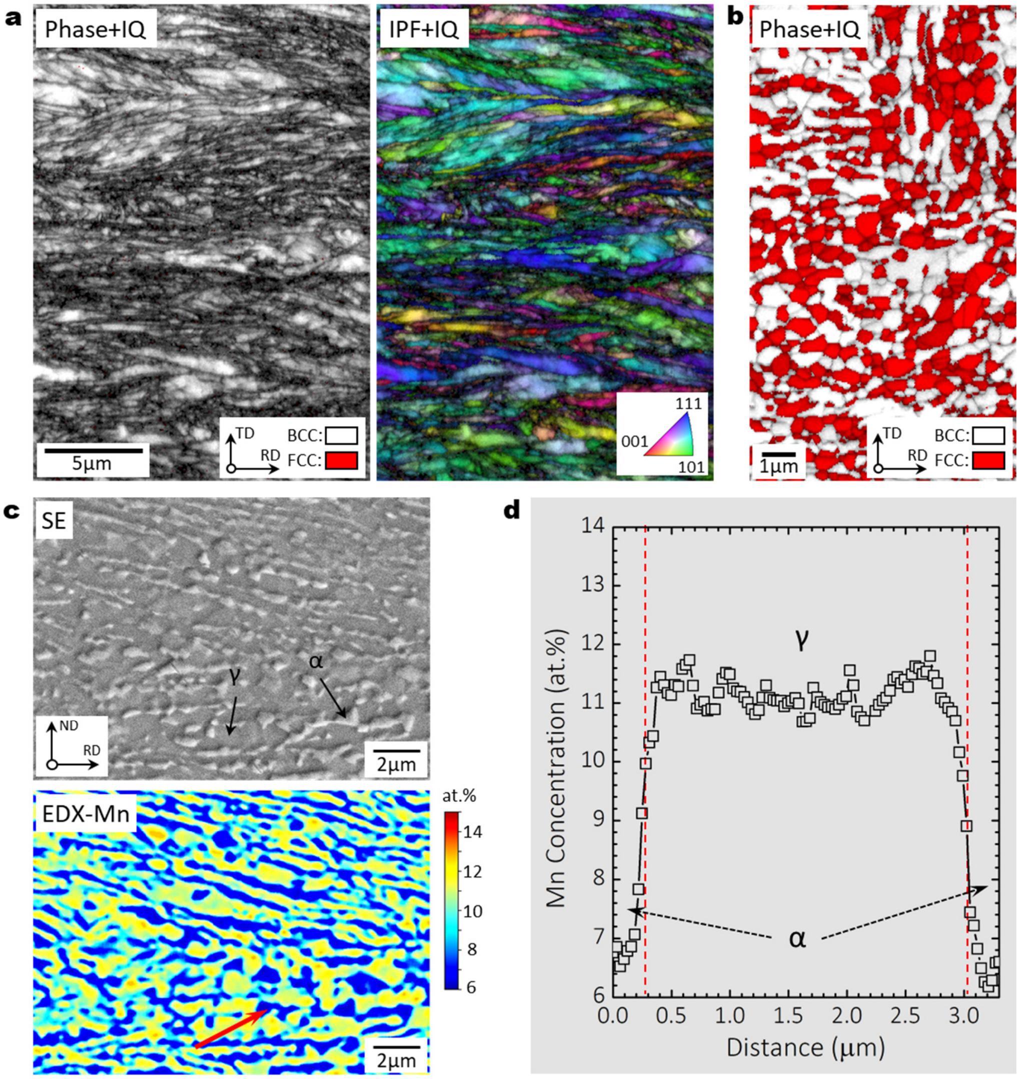

Supplementary Fig. 2 | Microstructure of the reference chemical homogeneous (HOM) sample before and after annealing. a, EBSD phase plus IQ map and inverse pole figure (IPF) plus IQ map of the sample in as-cold rolled condition, showing a fully martensitic microstructure. **b,** EBSD phase plus IQ map of the sample after heat treatment, showing an austenite-ferrite two-phase microstructure with a similar phase fraction and grain size as the HET sample (Supplementary Table 1). **c,** Secondary electron (SE) image and the corresponding energy-dispersive X-ray spectroscopy (EDX) area mapping of Mn for the same sample shown in **b**. **d,** Mn line profile crossing an austenite island, generated from the area marked by an arrow in **c**, showing a nearly homogenous distribution of Mn inside austenite with a concentration variation below 1 at.%.

Supplementary Table 1 | Detailed microstructural information of the HET and HOM samples. The phase fraction and grain size were measured by EBSD with a large probing area containing more than 10^4 grains.

Steel Sample	Volume Fraction		Grain Size	
	Austenite	Ferrite	Austenite	Ferrite
HET Sample	51.2%	48.8%	0.55 μm	0.53 μm
Reference HOM Sample	46.0%	54.0%	0.42 μm	0.54 μm

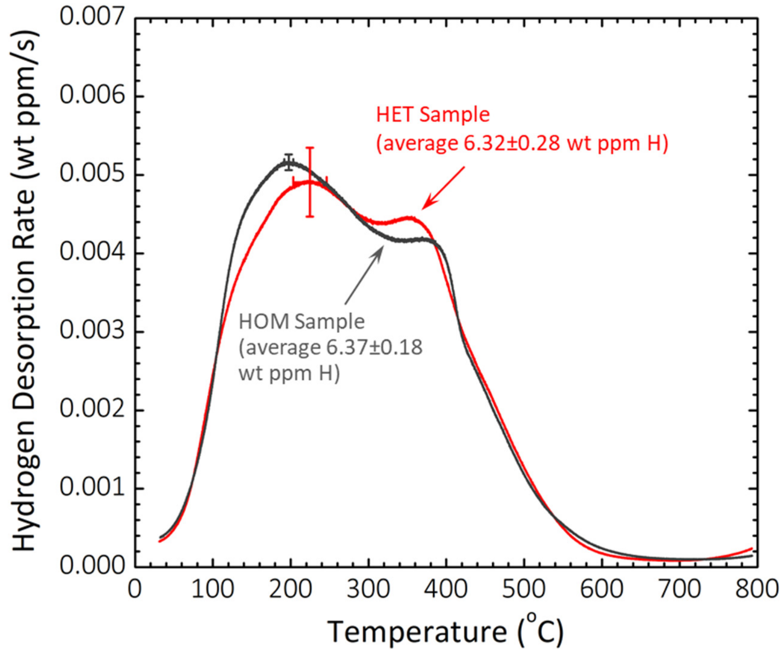

Supplementary Fig. 3 | Representative TDS results of the HET and HOM specimens pre-charged electrochemically for 24 h at a current density of 5 A/m^2. TDS experiments were conducted at a heating rate of 16 ˚C/min. The error bar represents the standard deviation determined from two and four repeated tests for the HOM and HET sample, respectively. The results show a very similar total H concentration for the two samples, treated at the same charging conditions. The similar shape of the two TDS spectra also suggests a similar H trapping/distribution behavior.

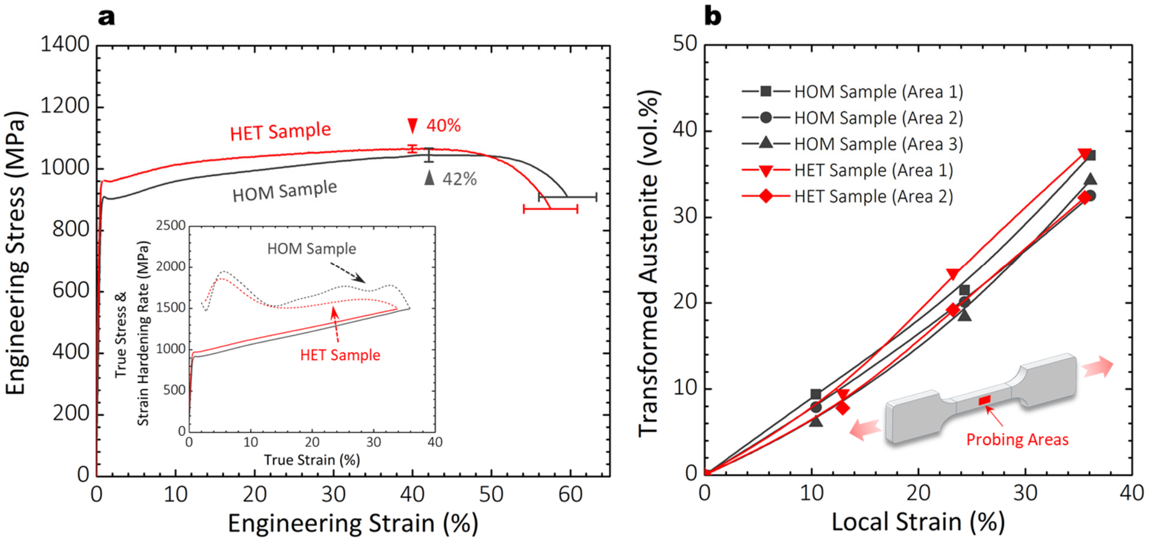

Supplementary Fig. 4 | Tensile property and deformation-induced austenite-to-martensite transformation behavior of the HET and reference HOM samples under H-free condition. **a,** Typical engineering stress-strain curve, with the strain rate response inset. The error bars represent the standard deviation from more than four repeating measurements. The point corresponding to the uniform strain/elongation for each tensile curve is marked by a triangular symbol and the strain value is added. **b,** The volume fraction of transformed austenite (equals the fraction of deformation-induced α'-martensite) as a function of local tensile strain, measured by *ex-situ* tensile testing combined with EBSD. Two local areas for the HET sample and three local areas for the HOM sample were selected for *ex-situ* probing. The size of each probing area is around 900 μm^2 covering more than 3500 grains, which should be large enough to reflect the global kinetics of martensite formation upon deformation. The overall kinetics of deformation-induced martensite formation is similar for the two samples up to the point of uniform elongation, meaning that the small volume fraction of $\gamma_{Mn\text{-}rich}$ (below 5 vol.%) in the HET sample does not influence the overall TRIP effect thus strain hardening and ductility.

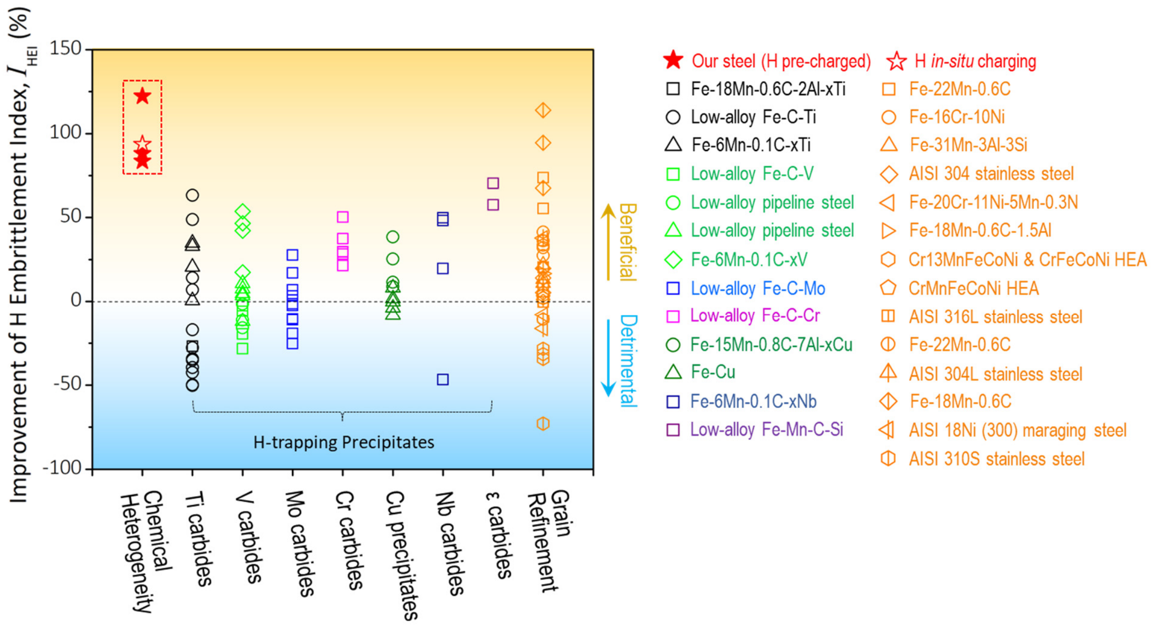

Supplementary Fig. 5 | Comparison between our microstructure engineering approach and other H-resistance enhancing methods reported in the literature, in terms of their effects on the improvement of the H embrittlement index (HEI). HEI is the ratio of ductility between the samples tested with H ($\varepsilon_{f\text{-}H}$) and without H ($\varepsilon_{f\text{-}0}$), i.e. $\varepsilon_{f\text{-}H}/\varepsilon_{f\text{-}0}$. This parameter has often been used to characterize the materials' resistance to H embrittlement [3]. The improvement of HEI (I_{HEI}) thus equals $(HEI_{after}\text{-}HEI_{before})/HEI_{before}\times100\%$, where HEI_{before} and HEI_{after} are the values before and after applying the specific approach, respectively. Same to the current study, the specimens before and after microstructure tuning reported in the literature were subjected to the same H charging condition (or a similar total H concentration) and the same testing conditions. Detailed information of these literature data regarding the values, material and testing conditions and references is listed in Supplementary Table 2.

Supplementary Table 2 | Details of the data presented in Fig. 2b and Supplementary Fig. 5. The ductility of all the materials in this table was measured by tensile tests on smooth specimens, subjected to a strain rate below ~10^{-3} s^{-1}. ($\varepsilon_{f\text{-}0}$: fracture strain/total elongation in H-free condition, $\varepsilon_{f\text{-}H}$: fracture strain in the presence of H, EC: electrochemical, RT: room temperature, as-Q: as-quenched state, Q&T: quenched and tempered, Q&P: quenched and partitioned, HEA: high-entropy alloys, PAGS: prior-austenite grain size).

HE mitigating approach	Material (composition in wt.%)	H-charging condition	Before applying the approach (%)			After applying the approach (%)			I_D (%)	I_{HEI} (%)	Refer -ence
			Sample condition	$\varepsilon_{f\text{-}0}$	$\varepsilon_{f\text{-}H}$	Sample condition	$\varepsilon_{f\text{-}0}$	$\varepsilon_{f\text{-}H}$			
Chemical heterogeneity	Fe-10Mn-0.2C-3Al-1Si	EC pre-charging (0.5 h-RT)	HOM	54.5 (±3.6)	22.2 (±1.5)	HET	52.6 (±3.4)	40.3 (±1.4)	81.5	88.1	This work
		EC pre-charging (24 h-RT)	HOM	54.5 (±3.6)	17.3 (±2.1)	HET	52.6 (±3.4)	30.7 (±1.7)	77.2	83.6	
		EC pre-charging (48 h-RT)	HOM	54.5 (±3.6)	10.3 (±3.6)	HET	52.6 (±3.4)	22.1 (±0.8)	114.6	122.3	
		EC *in-situ* charging (RT)	HOM	45.9* (±5.1)	6.2* (±0.6)	HET	45.1* (±6.8)	11.8* (±1.3)	90.3	93.6	
Ti-carbides precipitation	Fe-18Mn-0.6C-2Al-xTi	EC pre-charging (60 h-RT)	No Ti addition	70.9	68.9	0.15 wt.% Ti added	61.7	43.9	-36.3	-26.8	4
	Fe-0.1C-0.38Ti	EC pre-charging (1 h) + *in-situ* charging (RT)	As-Q (small amount of precipitates)	5.3	4.4	Q&T for 1 h	5.9	2.9	-32.6	-39.5	5*
				5.3	4.4	Q&T for 2 h	4.2	2.0	-54.1	-42.6	
				5.3	4.4	Q&T for 10 min	5.6	3.8	-11.9	-17.1	
	Fe-0.2C-0.74Ti		As-Q (small amount of precipitates)	5.4	4.2	Q&T for 1 h	6.0	2.4	-44.2	-49.8	
				5.4	4.2	Q&T for 2 h	4.3	1.7	-59.6	-50.2	
				5.4	4.2	Q&T for 10 min	5.7	3.0	-30.0	-34.3	
	Fe-0.3C-1.34Ti		As-Q (small amount of precipitates)	5.7	2.7	Q&T for 1 h	6.2	2.1	-21.7	-27.7	
				5.7	2.7	Q&T for 2 h	4.8	1.5	-44.9	-35.3	
				5.7	2.7	Q&T for 10 min	5.9	3.0	10.5	6.9	
		EC pre-charging to yield a total H of 6.6 wt ppm + *in-situ* charging (RT)	As-Q (small amount of precipitates)	5.7	2.7	Q&T for 1 h	6.2	4.7	76.8	63.3	
				5.7	2.7	Q&T for 2 h	4.8	3.4	26.6	48.8	
				5.7	2.7	Q&T for 10 min	5.9	3.2	18.0	14.2	
	Fe-0.1C-6Mn-xTi	EC pre-charging (1 h-RT)	No Ti addition	17.9	6.1	0.01 wt.% Ti added	16.5	6.8	11.0	20.5	6
		EC pre-charging (3 h-RT)		17.9	3.3		16.5	4.1	24.2	34.9	
		EC pre-charging (1 h-RT)		17.9	6.1	0.05 wt.% Ti added	13.7	4.7	-22.8	0.6	
		EC pre-charging (3 h-RT)		17.9	3.3		13.7	3.4	2.1	33.0	

V-carbides precipitation	Fe-0.1C-0.57V	EC pre-charging (1 h) + *in-situ* charging (RT)	As-Q (small amount of precipitates)	4.5	3.3	Q&T for 1 h	4.2	3	-9.1	-2.6	7*
	Fe-0.2C-1.09V			4.4	3.2		3.9	2.7	-16.6	-5.9	
	Fe-0.3C-1.67V			4.1	2.9		3.3	1.9	-34.5	-19.6	
				4.1	2.9	Q&T for 2 h	3.2	1.6	-44.3	-28.3	
	X80 pipeline steel	EC pre-charging (1 h)	No V addition	20.2	17.3	0.042 wt.% V added	24.8	17.9	3.3	-16.0	8*
				20.2	17.3	0.084 wt.% V added	26.1	19.9	14.5	-11.3	
				20.2	17.3	0.13 wt.% V added	27.1	22.9	32.4	-1.4	
	V-added X80 pipeline steel	EC pre-charging (1 h)	Tempered at 450˚C (small amount of precipitate)	22.0	17.5	Tempered at 500˚C	21.2	17.5	-0.2	3.5	9*
				22.0	17.5	Tempered at 550˚C	21.3	17.8	1.7	4.9	
				22.0	17.5	Tempered at 600˚C	20.4	17.5	-0.2	7.6	
				22.0	17.5	Tempered at 650˚C	20.6	18.2	3.8	10.8	
				22.0	17.5	Tempered at 700˚C	18.0	12.6	-27.9	-11.9	
	Fe-0.1C-6Mn-xV	EC pre-charging (1 h-RT)	No V addition	17.9	6.1	0.03 wt.% V added	15.4	7.7	26.2	46.5	6
		EC pre-charging (3 h-RT)		17.9	3.3		15.4	4.4	32.4	53.7	
		EC pre-charging (1 h-RT)		17.9	6.1	0.08 wt.% V added	14.5	5.8	-4.9	17.4	
		EC pre-charging (3 h-RT)		17.9	3.3		14.5	3.8	15.2	42.2	
Mo-carbides precipitation	Fe-0.1C-1.7Mo	EC pre-charging (1 h) + *in-situ* charging (RT)	As-Q (small amount of precipitates)	7.4	6.4	Q&T for 1 h	6.9	5.8	-9.4	-2.3	10*
				7.4	6.4	Q&T for 2 h	8.1	5.6	-12.1	-19.3	
		EC pre-charging to yield a total H of 2.1 wt ppm + *in-situ* charging (RT)		7.4	6.4	Q&T for 1 h	6.9	6.3	-0.8	7.0	
	Fe-0.14C-2.38Mo	EC pre-charging (1 h) + *in-situ* charging (RT)	As-Q (small amount of precipitates)	6.7	5.8	Q&T for 1 h	6.2	4.8	-17.5	-11.1	
				6.7	5.8	Q&T for 2 h	7.2	4.7	-19.7	-25.4	
		EC pre-charging to yield a total H of 2.9 wt ppm + *in-situ* charging (RT)		6.7	5.8	Q&T for 1 h	6.2	5.6	-4.1	3.3	
	Fe-0.18C-3.0Mo	EC pre-charging (1 h) + *in-situ* charging (RT), 5 mm/min crosshead speed	As-Q (small amount of precipitates)	6.9	6.7	Q&T for 1 h	7.5	7.2	6.9	-1.4	
				6.9	6.7	Q&T for 2 h	7.6	6.6	-2.2	-10.4	
		EC pre-charging (1 h) + *in-situ* charging (RT), 0.05 mm/min crosshead speed		6.6	3.8	Q&T for 1 h	7.2	5.2	39.2	27.7	
		EC *in-situ* charging (RT), 0.05 mm/min crosshead speed		6.6	4.4	Q&T for 1 h	7.2	5.6	28.0	17.1	
			As-Q	4.6	2.9	Q&T for 1 h	4.9	4.0	37.1	29.5	11*

Cr-carbides precipitation	Fe-0.1C-1.3Cr	EC pre-charging (1 h) + *in-situ* charging (RT)	(small amount of precipitates)	4.6	2.9	Q&T for 2 h	5.6	4.9	68.0	37.5	
	Fe-0.14C-1.8Cr		As-Q	4.5	2.8	Q&T for 1 h	3.7	3.0	7.3	29.6	
			(small amount of precipitates)	4.5	2.8	Q&T for 2 h	4.4	4.1	47.6	50.3	
	Fe-0.18C-2.2Cr		As-Q	4.5	3.0	Q&T for 1 h	3.0	2.5	-18.3	21.4	
			(small amount of precipitates)	4.5	3.0	Q&T for 2 h	3.6	3.1	4.3	28.2	
Introduction of Cu-based precipitates	Fe-15Mn-0.8C-7Al-xCu	EC pre-charging (1 mA/cm^2 & 48 h)	No Cu addition	39.6	30.4	1 wt.% Cu added	38.9	33.3	9.5	11.5	12
		EC pre-charging (3 mA/cm^2 & 48 h)		39.6	23.1		38.9	24.7	6.9	8.9	
		EC pre-charging (1 mA/cm^2 & 48 h)		39.6	30.4	3 wt.% Cu added	40.0	38.5	26.6	25.4	
		EC pre-charging (3 mA/cm^2 & 48 h)		39.6	23.1		40.0	32.3	39.8	38.4	
	Fe-1.7Cu	EC pre-charging (0.5 h) + *in-situ* charging (RT)	Solution treated	29.0	22.0	Aged for 10^2 s	23.4	19.2	-12.7	8.2	13
				29.0	22.0	Aged for 10^3 s	20.2	15.3	-30.5	-0.2	
				29.0	22.0	Aged for 10^4 s	16.3	11.9	-45.9	-3.8	
				29.0	22.0	Aged for 10^5 s	12.7	8.8	-59.9	-8.4	
				29.0	22.0	Aged for 10^6 s	12.8	9.9	-55.0	1.7	
Nb-carbides precipitation	Fe-0.1C-6Mn-xNb	EC pre-charging (1 h-RT)	No Nb addition	17.9	6.1	0.02 wt.% Nb added	15.2	6.2	1.6	19.7	6
		EC pre-charging (3 h-RT)		17.9	3.3		15.2	4.2	27.3	49.9	
		EC pre-charging (1 h-RT)		17.9	6.1	0.1 wt.% Nb added	13.9	7.0	14.8	48.2	
		EC pre-charging (3 h-RT)		17.9	3.3		13.9	1.4	-58.8	-46.8	
ε-carbides precipitation	Fe-1.8Mn-0.2C-1.4Si	EC pre-charging (1 min-RT)	Q&P	19.7	11.3	Q&P and tempered	18.0	17.6	55.8	70.5	14
		EC pre-charging (5 min-RT)		19.7	2.5		18.0	3.6	44.0	57.6	
Grain refinement	Fe-22Mn-0.6C	EC pre-charging (72 h-RT)	Grain size 45 μm	93	52.1	Grain size 20 μm	90.9	61.3	17.7	20.4	15*
		EC pre-charging (166 h-RT)		93	48.4		90.9	57	17.8	20.5	
		EC pre-charging (72 h-RT)		93	52.1	Grain size 7 μm	73.9	64.3	23.4	55.3	
		EC pre-charging (166 h-RT)		93	48.4		73.9	52.4	8.3	36.2	
		EC pre-charging (72 h-RT)		93	52.1	Grain size 4 μm	67.1	65.3	25.3	73.7	
		EC pre-charging (166 h-RT)		93	48.4		67.1	60.7	25.4	73.8	
	Fe-16Cr-10Ni	Gas pre-charging (10 MPa, 72 h-270°C)	Grain size 21 μm	66	51	Grain size 12.3 μm	64	66	29.4	33.5	16
				66	51	Grain size 9.6 μm	63	62	21.6	27.4	
				66	51	Grain size 6.3 μm	58	61	19.6	36.1	
				66	51	Grain size 5.8 μm	54	59	15.7	41.4	
				66	51	Grain size 1 μm	50	51	0.0	32.0	

	Fe-31Mn-3Al-3Si	EC pre-charging (48 h-RT)	Grain size 19 μm	117.9	97.8	Grain size 1.5 μm	69.3	63.8	-34.8	11.0	17*
				117.9	97.8	Grain size 0.58 μm	55.1	55.7	-43.0	21.9	
	AISI 304 stainless steel	EC *in-situ* charging (RT)	Grain size 12 μm	62.8	50.2	Grain size 8 μm	59.8	50.3	0.2	5.2	18*
				62.8	50.2	Grain size 4 μm	55.75	50.8	1.2	14.0	
	Fe-20Cr-11Ni-5Mn-0.3N	EC pre-charging (48 h-90 ˚C)	Grain size 63 μm	61.6	62.3	Grain size 23 μm	65.1	60.6	-2.7	-8.0	19
	Fe-18Mn-0.6C-1.5Al		Grain size 45 μm	82.2	64.3	Grain size 23 μm	77.6	70.8	10.1	16.6	
	CrMnFeCoNi HEA (13 at.% Mn)	Gas pre-charging (100 MPa, 200 h-270˚C)	Grain size 200 μm	55	22	Grain size 1.6 μm	49	20	-9.1	2.0	20
	CrFeCoNi HEA		Grain size 170 μm	54	50	Grain size 1.9 μm	51	49	-2.0	3.8	
	CrMnFeCoNi HEA	Gas pre-charging (100 MPa, 200 h-270˚C)	Grain size 22 μm	52.8	21.5	Grain size 1.9 μm	41	16.7	-22.3	0.0	21
			Grain size 22 μm	52.8	21.5	Grain size 1.5 μm	28.8	12	-44.2	2.3	
	AISI 316L stainless steel	EC pre-charging (144 h-95 ˚C)	Grain size 300 μm	57	49	Grain size 140 μm	70	60	22.4	-0.3	22
		EC pre-charging (5h-150 ˚C)		57	43		70	58	34.9	9.8	
	Fe-22Mn-0.6C	EC pre-charging (48 h-RT)	Grain size 21 μm	88.9	67.6	Grain size 0.58 μm	60.6	41.1	-39.2	-10.8	23
	AISI 304L stainless steel	Gas *in-situ* charging (40 MPa, 25˚C)	Grain size 130 μm	68	54	Grain size 13 μm	60	54	0.0	13.3	24
	Fe-18Mn-0.6C	EC pre-charging (48 h-RT)	Grain size 79.8 μm	73.9	32.8	Grain size 53.1 μm	64.9	34.5	5.2	19.8	25
				73.9	32.8	Grain size 30.4 μm	61.6	45.8	39.6	67.5	
				73.9	32.8	Grain size 16.5 μm	62.2	53.7	63.7	94.5	
				73.9	32.8	Grain size 6.4 μm	61.2	58.1	77.1	113.9	
	AISI 18Ni (300) maraging steel	EC *in-situ* charging (RT)	PAGS 520 μm (aged at 480 ˚C)	5.1	1.3	PAGS 28.6 μm (aged at 480 ˚C)	8.9	1.9	46.2	-16.2	26*
			PAGS 373 μm (aged at 560 ˚C)	6.3	2	PAGS 30 μm (aged at 560 ˚C)	6.4	2.8	40.0	37.8	
	AISI 310S stainless steel	Gas pre-charging (10 MPa, 160 h-270˚C)	Grain size 28 μm	53.2	54.8	Grain size 0.27 μm	14.7	9.9	-81.9	-34.4	27
				53.2	54.8	Grain size 0.21 μm	14.5	10.7	-80.5	-28.4	
				53.2	54.8	Grain size 0.17 μm	12.9	9.1	-83.4	-31.6	
				53.2	54.8	Grain size 0.15 μm	10.5	2.9	-94.6	-72.9	
				53.2	54.8	Grain size 0.09 μm	10.5	9.7	-82.3	-10.6	

*Values determined from tensile testing without the use of extensometer (i.e. the absolute ductility value is overestimated).

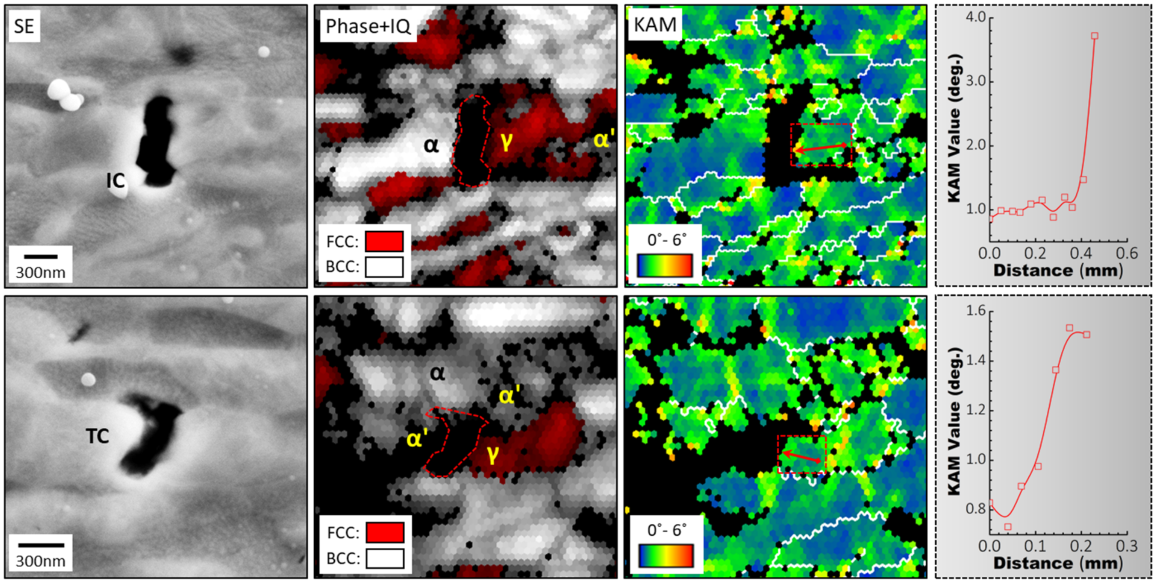

Supplementary Fig. 6 | SE imaging and EBSD analysis (phase plus IQ map and kernel average misorientation (KAM) map) on representative H-induced cracks in the HET sample pre-charged with ~6.5 wt ppm H. One typical intergranular crack (IC) and one transgranular crack (TC) are presented. Differentiating ferrite and α'-martensite was based on the intensity of the EBSD IQ map and KAM map. The profile of the KAM is acquired from the region marked by the red arrow in the corresponding KAM map on the left hand side. A high KAM value suggests a high number of geometrically necessary dislocations, meaning a high plastic deformation. The cracks are blunted due to the strong plastic compliance of untransformed stable austenite in the Mn-rich regions.

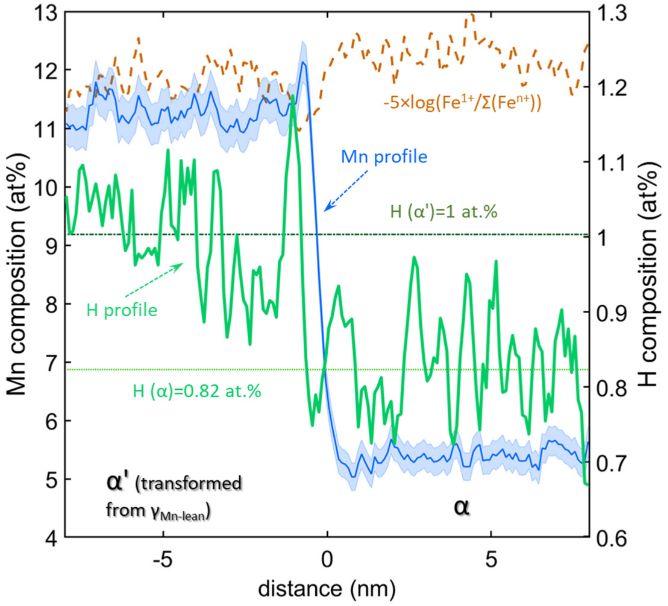

Supplementary Fig. 7 | H and Mn profiles across the ferrite and deformation-induced martensite interface displayed in the APT dataset in Fig. 4c. The log of the ratio of Fe^{1+} to all charge states is also plotted in dark orange along with the profiles. The peak of H at the interface is attributed to an actual segregation of H at the α-α' phase boundary, no matter whether the H comes from electrochemical charging or from focused-ion beam preparation (Supplementary Note 2). This observation reveals that H tends to segregate to hetero-interfaces which are associated with martensite. This H segregation promotes interface decohesion and thus also crack nucleation at α-α' phase boundaries as observed in Fig. 4b.

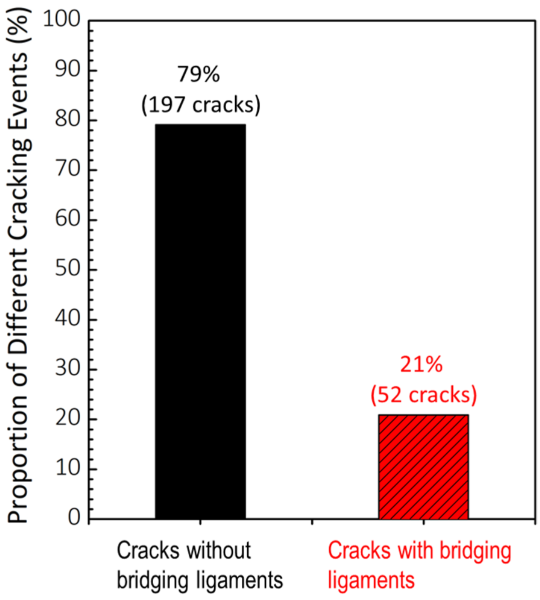

Supplementary Fig. 8 | Proportions of microcracks with and without bridging ligaments in the fractured HET sample pre-charged with ~6.5 wt ppm H. All the H-induced microcracks adjacent to the fracture surface were probed and statistically analyzed. Among the analyzed ~250 microcracks, the ones containing bridging ligaments constitute ~21%. These results suggest that the crack bridging effect, due to the stable Mn-rich austenite forming ductile ligaments, plays a distinct but minor role in enhancing the overall fracture resistance (in the presence of H) compared with the microcrack blunting/arresting effect occurring at the crack front (or crack tip).

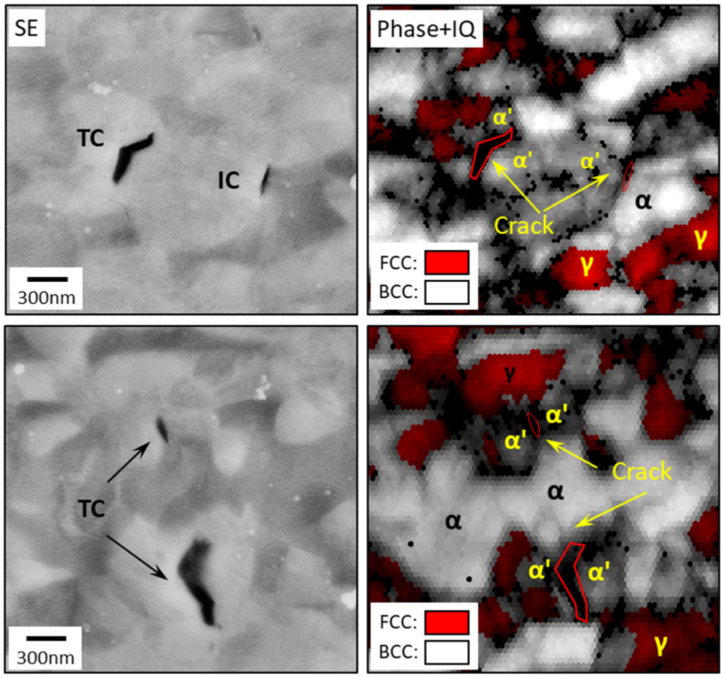

Supplementary Fig. 9 | SE imaging and EBSD analysis (phase plus IQ map) on representative H-induced intergranular cracks (IC) and transgranular cracks (TC) in the reference HOM sample pre-charged with ~6.5 wt ppm H. All the austenite regions adjacent to the cracks have transformed into α'-martensite. This phenomenon is observed for all the cracks we have probed using SE and EBSD (in total around 30 cracks). It supports that in this sample, the austenite phase near the crack tips is readily transformed to martensite due to high stress/strain concentrations at these regions.

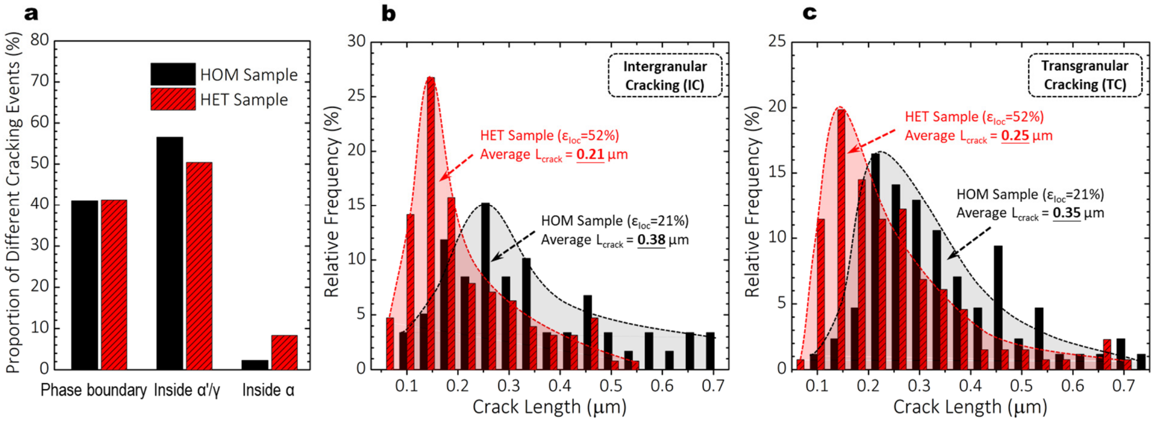

Supplementary Fig. 10 | Statistical analysis of H-induced cracks in the fractured HET and HOM samples pre-charged with ~6.5 wt ppm H. a, Proportion of different H-induced cracking events, showing that in both types of materials, H-induced cracks mainly form at ferrite/strain-induced α'-martensite interfaces (intergranular type) and inside the α'-martensite (transgranular type). Note that even for the case of transgranular cracking, the cracks are still preferably nucleated at the α-α' interfaces (as shown in Fig. 4b), followed by their propagation into α'-martensite. **b, c,** Crack length (L_{crack}) distribution for the intergranular cracks and transgranular cracks, respectively. The crack length, regardless of their crack formation sites, appears much shorter in the HET sample compared with the HOM sample, even though the local strain (ε_{loc}) in the probed region is much larger in the former specimen (52% for the HET sample vs. 21% for the HOM sample, measured by digital image correlation (DIC)). The results are acquired from ~150 and ~250 probed cracks for the HOM and HET samples, respectively.

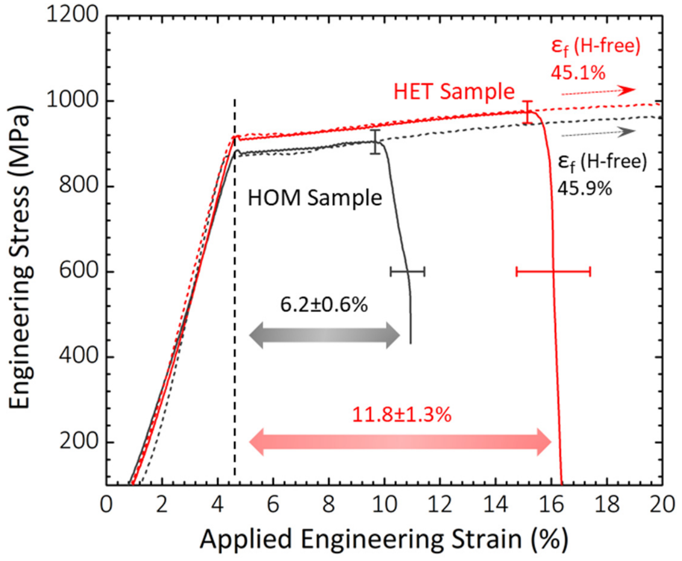

Supplementary Fig. 11 | Slow strain rate tensile testing results for samples under *in-situ* electrochemical H charging. Since no extensometer can be used here, the strain was calculated by the crosshead displacement. Due to the inaccuracy of the elastic part, the ductility was quantified by the plastic strain at fracture (fracture strain, ε_f, minus the strain at the yield point). The two samples show a similar total elongation ~45% and uniform elongation ~40% when tested under air (dash curves). The error bars represent the standard deviation from more than two repeating experiments.

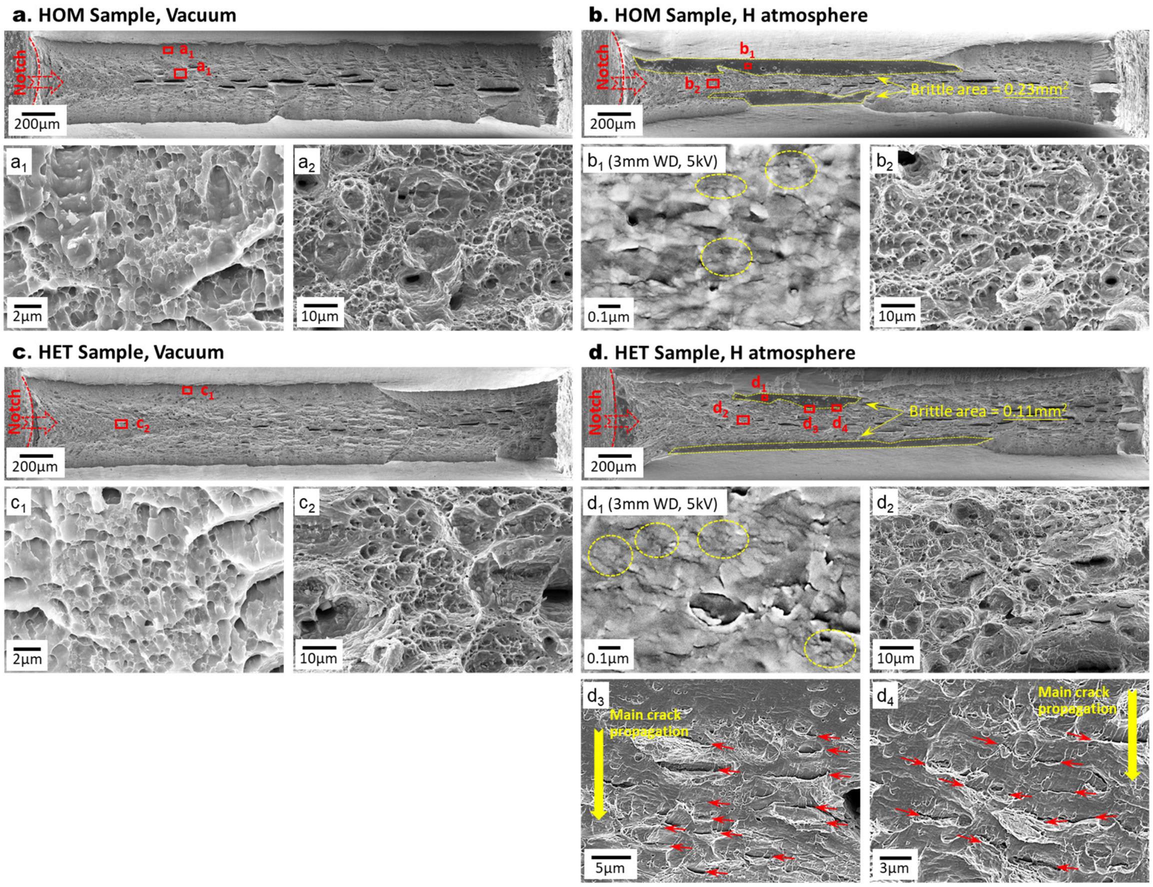

Supplementary Fig. 12 | Fracture surface analysis of samples deformed under vacuum inside the SEM chamber and under H atmosphere (*in-situ* H-plasma charging). a, b, Reference HOM sample fractured under vacuum and under H exposure, respectively. **c, d,** The HET sample fractured under vacuum and H atmosphere, respectively. **a_1~a_2, b_1~b_2, c_1~c_2** and **d_1~d_4** are magnified images taken from the rectangular frames in **a, b, c** and **d**, respectively. Under vacuum, both samples show a dimpled fracture behavior throughout the whole fracture surface (the dimples near the sample surface are sheared). Under H atmosphere, the fracture surface of both samples contains two types of regions with different fracture features. Region 1: Brittle region near the sample surfaces, which is characterized by a smooth, large facet at low magnifications (see the overview images). However, when magnifying these regions and probing with very low working distance (WD, 3 mm) and accelerating voltage (5 kV) to achieve a better surface resolution, we observe very small facets (~100 nm) and nanoscaled "mottled", dark-bright contrast in some regions (see elliptical frames in **b_1** and **d_1**). The latter feature was proposed to be a result of dense coverage of "nano-dimples" [28], which have been formed due to a combined effect of H-enhanced localized plasticity (HELP) and H-enhanced strain-induced superabundant vacancies (HESIV) [28-30]. The occurrence of the HELP mechanism in both types

of samples is supported by the smaller cross-sectional area of the fracture surface when deformed under H atmosphere compared with that loaded under vacuum. Region 2: Dimpled ductile region away from the specimen surface (see $\mathbf{b_2}$ and $\mathbf{d_2}$). It is clear that Region 1 is caused by the formation and propagation of H-induced cracks. Thus the size of the brittle region should be equal to the size of the main H-induced crack, which is observed to be much smaller for the HET sample compared to that for the HOM sample (0.11 mm^2 vs. 0.23 mm^2). This is due to (a) the suppression effect of Mn-rich stable austenite on H ingress/penetration during deformation when most metastable austenite has transformed to α'-martensite (when the total austenite fraction is below 10 vol.% [31]) and (b) the strong blocking effect of the Mn-rich buffer austenite on H-induced cracks with mechanisms shown in Fig. 4a. These two factors effectively slow the main crack propagation velocity down. We observe a high density of small secondary cracks or steps in the fracture surface taken at the brittle-ductile transition regions in the HET sample (see arrows in $\mathbf{d_3}$ and $\mathbf{d_4}$), whereas these features are absent in the HOM sample. These features are formed due to the presence of the high-density of $\gamma_{Mn\text{-}rich}$ which act as obstacles frequently blocking or deflecting cracks.

Supplementary Note 1 – Role of the different processing steps for the microstructure evolution

The austenite's heterogeneous Mn distribution in this work was realized through three processing steps after hot rolling. The first intermediate annealing conducted at a temperature (700˚C) just above the Ac1 temperature allows the formation of some fraction of dispersed austenite ($\gamma_{Mn\text{-}rich}$) with a high Mn enrichment (up to 16 wt.%) due to strong elemental partitioning [32]. The second cold rolling process has two aims: (a) it partially transforms $\gamma_{Mn\text{-}rich}$ to α'-martensite, accelerating the reversion of new austenite during the final annealing step [33]; (b) the partial austenite-to-martensite transformation fragments the $\gamma_{Mn\text{-}rich}$ zones, thereby increasing its number density and dispersion. The microstructure after this processing step is shown in Supplementary Fig. 1a. The final intercritical annealing performed at a relatively high temperature (750 ˚C, 50 ˚C higher than the preceding intermediate annealing temperature) enables the reversion of new austenite from martensite and ferrite. The new austenite formed at this stage contains a lower Mn content ($\gamma_{Mn\text{-}lean}$), as shown from the DICTRA simulation results in Supplementary Fig. 1b.

Supplementary Note 2 – H segregation at the interface between ferrite and α'-martensite

Here we discuss the H segregation behavior at the interface between ferrite and deformation-induced α'-martensite revealed in the APT results. An isocomposition surface with a threshold of 6 at.% Mn was first superimposed on the point cloud obtained from the dataset displayed in Fig. 4c, in order to delineate the interface between ferrite and deformation-induced martensite (transformed from $\gamma_{Mn\text{-}lean}$). A composition profile in the form of a proximity histogram was then calculated to estimate the composition near the interface [34]. The profile of Mn and H are plotted in Supplementary Fig. 7 in blue and green, respectively. There appears to be a slight segregation of Mn at the interface, but also a peak of H. The two horizontal lines correspond to the average composition in H in α and α', i.e. 0.82 at.% and 1 at.% respectively, and the peak composition, at the interface, is approx. 1.2 at.%.

Measuring the composition of H within APT datasets is extremely challenging and typically avoided, unless isotopic labelling is used [35,36]. Indeed, deuteration helps avoid overlap with the residual H from the vacuum chamber, which is typically found to affect data in regions of the specimen where the electrostatic field is low. However, recent studies have shown that specimen preparation by focused-ion beam leads to an uncontrolled incorporation of H within the specimen [36-38], which might be used as a qualitative assessment of the trapping/segregation

behavior of H atoms. Interpreting variations in the measured H composition requires a careful assessment of the changes in the intensity of the electrostatic field used to trigger the field evaporation during the APT analysis [36]. Within the dataset (Fig. 4c in the manuscript), Fe appears in the 1+ and 2+ charge states, and the ratio of the charge states give qualitative information on the local changes in the electrostatic field, as studied in details by Kingham [39]. The log of the ratio of Fe^{1+} to all charge states is plotted in dark orange along with the profile in Supplementary Fig. 7. Based on this data, we can see that the electrostatic field is slightly higher in α' than in α, and it drops slightly at the interface. Based on the recalculated Kingham curves in Ref. [40], the difference in the electric field between the two phases is around 2%. The higher amount of H in α', i.e. where the field is higher, can hence be safely interpreted as an indication that the H is effectively more highly concentrated in this phase [41], as can be expected due to the high dislocation density inside α'. Since the changes in the electrostatic field are very low, the peak of H at the interface can be attributed to an actual segregation of H at the α-α' phase boundary, no matter whether the H comes from electrochemical charging or from focused-ion beam. Such observation validates that H tends to segregate at martensite associated hetero-interfaces which promotes interface decohesion as shown in Fig. 4b and Supplementary Fig. 10a.

Supplementary Note 3 – Difference between H pre-charging and *in-situ* H charging in terms of H ingress, distribution and H embrittlement mechanisms

For the investigated samples deformed under *in-situ* H charging conditions, H ingress occurs concurrently with the deformation-induced phase transformation from austenite to α'-martensite. Since the H diffusivity in α'-martensite is around 2~5 orders of magnitude higher than that in austenite [42-45], we expect a higher rate of H ingress at high strain levels, especially when most of the austenite has transformed to martensite and when high strain/stress is locally concentrated (e.g. near the tip of propagating cracks). This accelerated H ingress at later deformation stages provides important information for interpreting the mechanical and failure behavior of samples exposed to *in-situ* H-plasma charging. The partial pressure of the plasma phase in the current setup is about 40 Pa. This is a relatively low pressure in comparison with electrochemical cathodic charging (can reach tens of MPa of H fugacity [46]). The H uptake is thus limited before sufficient austenite-to-martensite transformation occurs, which is the reason why a measurable difference in the macroscopic tensile curves between uncharged and *in-situ* H-plasma charged specimens has not been detected. However, the fracture surfaces shown in Supplementary Fig. 12 reveal a distinct embrittlement effect, which can then be attributed to the

accelerated H ingress during the macroscopic crack propagation stage. In addition, the behavior of H trapping and internal migration upon H pre-charging or *in-situ* charging should also be very different. For example, the initial microstructure of the investigated steel has a large area fraction of austenite-ferrite phase boundaries, which are strong H trapping sites (H binding energy ~50 kJ/mol [47]). However, such type of hetero-interface gradually disappears during deformation due to deformation-driven austenite-to-martensite transformation. This means that a high amount of H can be trapped at austenite-ferrite phase boundaries after H pre-charging [47], but this will not occur for the case of *in-situ* charging if H is mainly introduced at high inelastic deformation levels. The different behaviors of H ingress, trapping and migration could change the prevalent H embrittlement mechanisms, namely, from a dominant H-enhanced decohesion (HEDE) at hetero-interfaces [47] (Supplementary Figs. 7 and 10a) for the case of H pre-charging, to a combined H-enhanced localized plasticity (HELP) and H-enhanced strain-induced vacancy (HESIV) mechanism (Supplementary Fig. 12) for the case of *in-situ* H-plasma charging. For either case, we demonstrate here, regardless of how and when H is introduced and what the specific operating H embrittlement mechanism is, that our approach of producing chemical heterogeneity always enhances the materials' resistance to H embrittlement (Fig. 2 and Supplementary Figs. 11 and 12). In comparison to the role of $\gamma_{Mn\text{-}rich}$ in enhancing the alloy's H-resistance in pre-charged samples (i.e. suppressing deformation-induced internal H migration as well as microcrack propagation), the architected $\gamma_{Mn\text{-}rich}$ zones have one additionally beneficial effect in resisting H environmental embrittlement, that is, they can also suppress the H ingress or penetration during deformation due to their trapping effect. This effect is reported to be more pronounced when most metastable austenite has transformed to martensite (i.e. the total austenite fraction achieves below 10 vol.% [31]).

Supplementary Note 4 – Implication of the achieved H-resistance improvement on future application of the materials

In this work, we have shown that the investigated steel, after applying the chemical heterogeneity strategy, achieves a much higher resistance to H embrittlement (by a factor of two as shown in Fig. 2a), clearly surpassing that of other commercial high-strength steels with a similar strength level, regardless of how the H embrittlement resistance is characterized (i.e. either by the absolute ductility in the presence of H or by the H embrittlement index, see Supplementary Fig. 13). Given that these commercial steels have already been used in the automotive industry (e.g. safety-critical parts like B-pillar reinforcement), we expect that the

chemical heterogeneity manipulated (HET) material should also match and even surpass the required standards for final application in corresponding application fields such as for automotive parts. In order to further support this view, we performed additional constant loading experiments based on the SEP 1970 and VDA 238-202 standard testing protocols [48,49], which are globally used to validate the safe use of high-strength steels in the automotive industry. Results show that at the 100% macroscopic yield strength level within the 5% NaCl aqueous solution, the chemical homogeneous (HOM) sample fractured at around 19.9 hours, whereas the HET sample did not fail after 96 hours (also no formation of macroscopic cracks was observed) and thus fully meets and surpasses the automotive industry standards for commercial application [48-51]. These results mark a valuable progress made by our H resistance-enhancing strategy in promoting the successful applications of high-strength steels in corresponding application fields.

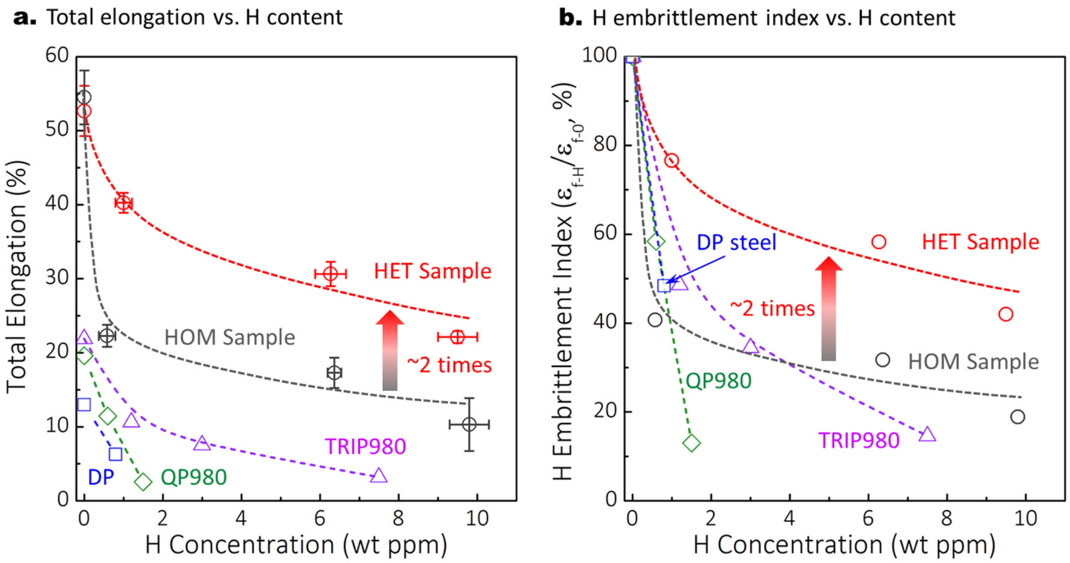

Supplementary Fig. 13 | Comparison of the H embrittlement resistance between the presented two steel samples (HET and HOM) and other commercial advanced high-strength steels with a similar strength level (i.e. yield strength above ~600 MPa and tensile strength of ~1000 MPa), including DP [44], QP980 [52] and TRIP980 [53] steels. The change of **a,** absolute ductility and **b,** the H embrittlement index as a function of total H concentration. **a** is also included in Fig. 2b (as the inset). Note that the H uptake in automotive/construction applications is often due to cathodic and corrosion reactions and is typically of the order of a few wt ppm [50,54-56].

Like many other damage-tolerant methods for improving materials' overall ductility/toughness [57], our approach of designing microstructures with chemical heterogeneity in principle does not suppress the nucleation of microcracks. However, we find, in the HET sample,

that all the H-induced cracks nucleated within the uniform deformation regime can be successfully arrested and remain confined to a size of only a few micrometers (as demonstrated in Supplementary Table 3), thus rendering them harmless. The presence of such microscale damage features, formed due to the presence of H at certain deformation stages, should not influence the materials' performance when used for industry applications, as all medium- and high-strength engineering alloys (e.g. steels and Al alloys) generally inevitably contain multiple micro-damage features that are inherited from the preceding casting and forming processes [58].

Supplementary Table 3 | The influence of H on the density, size and area fraction of deformation-induced damage features in the HET sample. The density and area fraction of damage features formed within the macroscopic uniform deformation regime are in our material not significantly increased by the presence of H and should be lower than the damaging extent that a typical engineering safety-critical part can tolerate. All the nucleated H-induced cracks were observed to be arrested and remain at a size below ~3.6 μm.

Specimen	Probing Area	Damage Density	Damage Size*	Damage Area Fraction
HET sample, H charged (~6.5 wt ppm H) and deformed to its uniform strain level (~30%)**	0.57×1.7 mm^2	179 mm^{-2}	< ~3.6 μm	0.021%
HET sample, non-charged and deformed to ~30% strain	0.57×1.3 mm^2	108 mm^{-2}	< ~1.8 μm	0.019%

*Here the damage size refers to the length of cracks or the length of the elliptical voids nucleated within the metal matrix.
**Note that the damage analysis in the H-charged specimen was conducted very close to the sample surface where the H concentration is the highest and cracks are easiest to form.

Supplementary References

1. Wei, R., Enomoto, M., Hadian, R., Zurob, H. & Purdy, G. Growth of austenite from as-quenched martensite during intercritical annealing in an Fe-0.1 C-3Mn-1.5 Si alloy. *Acta Materialia* **61**, 697-707 (2013).
2. Ding, R. *et al.* Effect of pre-existed austenite on austenite reversion and mechanical behavior of an Fe-0.2 C-8Mn-2Al medium Mn steel. *Acta Materialia* **147**, 59-69 (2018).
3. Lee, J. A. Hydrogen Embrittlement of Nickel, Cobalt and Iron-based Superalloys. in *Gaseous Hydrogen Embrittlement of Materials in Energy Technologies* (ed. Gangloff, R. P. and Somerday, B. P.) 624-667 (Woodhead Publishing Limited, Cambridge, 2012).
4. Park, I.-J., Jo, S. Y., Kang, M., Lee, S.-M. & Lee, Y.-K. The effect of Ti precipitates on hydrogen embrittlement of Fe-18Mn-0.6 C-2Al-xTi twinning-induced plasticity steel. *Corrosion Science* **89**, 38-45 (2014).
5. Depover, T. & Verbeken, K. The effect of TiC on the hydrogen induced ductility loss and trapping behavior of Fe-C-Ti alloys. *Corrosion Science* **112**, 308-326 (2016).
6. Park, T. M., Kim, H.-J., Um, H. Y., Goo, N. H. & Han, J. The possibility of enhanced hydrogen embrittlement resistance of medium-Mn steels by addition of micro-alloying elements. *Materials Characterization* **165**, 110386 (2020).
7. Depover, T. & Verbeken, K. Evaluation of the effect of V_4C_3 precipitates on the hydrogen induced mechanical degradation in Fe-C-V alloys. *Materials Science and Engineering: A* **675**, 299-313 (2016).
8. Li, L., Song, B., Cai, Z., Liu, Z. & Cui, X. Effect of vanadium content on hydrogen diffusion behaviors and hydrogen induced ductility loss of X80 pipeline steel. *Materials Science and Engineering: A* **742**, 712-721 (2019).
9. Li, L., Song, B., Yang, B., Wang, L. & Cheng, W. Effect of tempering temperature after thermo-mechanical control process on microstructure characteristics and hydrogen-induced ductility loss in high-vanadium X80 pipeline steel. *Materials* **13**, 2839 (2020).
10. Depover, T. & Verbeken, K. Evaluation of the role of Mo_2C in hydrogen induced ductility loss in Q&T Fe-C-Mo alloys. *International Journal of Hydrogen Energy* **41**, 14310-14329 (2016).
11. Depover, T. & Verbeken, K. Hydrogen trapping and hydrogen induced mechanical degradation in lab cast Fe-C-Cr alloys. *Materials Science and Engineering: A* **669**, 134-149 (2016).
12. Yoo, J. *et al.* Effects of Cu addition on resistance to hydrogen embrittlement in 1 GPa-grade duplex lightweight steels. *Acta Materialia* **196**, 370-383 (2020).
13. Takano, N., Yokka, Y. & Terasaki, F. The effect of copper precipitation on hydrogen embrittlement in iron. *Materials Science and Engineering: A* **387**, 428-432 (2004).
14. Zhu, X. *et al.* Improved resistance to hydrogen embrittlement in a high-strength steel by quenching-partitioning-tempering treatment. *Scripta Materialia* **97**, 21-24 (2015).
15. Zan, N., Ding, H., Guo, X., Tang, Z. & Bleck, W. Effects of grain size on hydrogen embrittlement in a Fe-22Mn-0.6 C TWIP steel. *International Journal of Hydrogen Energy* **40**, 10687-10696 (2015).
16. Macadre, A., Nakada, N., Tsuchiyama, T. & Takaki, S. Critical grain size to limit the hydrogen-induced ductility drop in a metastable austenitic steel. *International Journal of Hydrogen Energy* **40**, 10697-10703 (2015).
17. Bai, Y., Momotani, Y., Chen, M., Shibata, A. & Tsuji, N. Effect of grain refinement on hydrogen embrittlement behaviors of high-Mn TWIP steel. *Materials Science and Engineering: A* **651**, 935-944 (2016).
18. Fan, Y., Zhang, B., Wang, J., Han, E.-H. & Ke, W. Effect of grain refinement on the hydrogen embrittlement of 304 austenitic stainless steel. *Journal of Materials Science & Technology* **35**, 2213-2219 (2019).

19. Noh, H.-S., Kang, J.-H. & Kim, S.-J. Effect of grain size on hydrogen embrittlement in stable austenitic high-Mn TWIP and high-N stainless steels. *International Journal of Hydrogen Energy* **44**, 25076-25090 (2019).
20. Koyama, M., Wang, H., Verma, V. K., Tsuzaki, K. & Akiyama, E. Effects of Mn content and grain size on hydrogen embrittlement susceptibility of face-centered cubic high-entropy alloys. *Metallurgical and Materials Transactions A* **51**, 5612-5616 (2020).
21. Koyama, M., Ichii, K. & Tsuzaki, K. Grain refinement effect on hydrogen embrittlement resistance of an equiatomic CoCrFeMnNi high-entropy alloy. *International Journal of Hydrogen Energy* **44**, 17163-17167 (2019).
22. Brass, A.-M. & Chêne, J. Hydrogen uptake in 316L stainless steel: consequences on the tensile properties. *Corrosion Science* **48**, 3222-3242 (2006).
23. Bai, Y., Tian, Y., Gao, S., Shibata, A. & Tsuji, N. Hydrogen embrittlement behaviors of ultrafine-grained 22Mn-0.6 C austenitic twinning induced plasticity steel. *Journal of Materials Research* **32**, 4592-4604 (2017).
24. Weber, S., Martin, M. & Theisen, W. Impact of heat treatment on the mechanical properties of AISI 304L austenitic stainless steel in high-pressure hydrogen gas. *Journal of Materials Science* **47**, 6095-6107 (2012).
25. Park, I.-J., Lee, S.-m., Jeon, H.-h. & Lee, Y.-K. The advantage of grain refinement in the hydrogen embrittlement of Fe-18Mn-0.6 C twinning-induced plasticity steel. *Corrosion Science* **93**, 63-69 (2015).
26. Gomes da Silva, M. J. *et al.* The effect of prior austenite grain size on hydrogen embrittlement of Co-containing 18Ni 300 maraging steel. *International Journal of Hydrogen Energy* **44**, 18606-18615 (2019).
27. Mine, Y., Tachibana, K. & Horita, Z. Effect of hydrogen on tensile properties of ultrafine-grained type 310S austenitic stainless steel processed by high-pressure torsion. *Metallurgical and Materials Transactions A* **42**, 1619-1629 (2011).
28. Neeraj, T., Srinivasan, R. & Li, J. Hydrogen embrittlement of ferritic steels: observations on deformation microstructure, nanoscale dimples and failure by nanovoiding. *Acta Materialia* **60**, 5160-5171 (2012).
29. Nagumo, M. & Takai, K. The predominant role of strain-induced vacancies in hydrogen embrittlement of steels: overview. *Acta Materialia* **165**, 722-733 (2019).
30. Robertson, I. The effect of hydrogen on dislocation dynamics. *Engineering Fracture Mechanics* **64**, 649-673 (1999).
31. Fielding, L., Song, E. J., Han, D.-K., Bhadeshia, H. & Suh, D.-W. Hydrogen diffusion and the percolation of austenite in nanostructured bainitic steel. *Proceedings of the Royal Society A: Mathematical, Physical and Engineering Sciences* **470**, 20140108 (2014).
32. Sun, B. *et al.* Microstructural characteristics and tensile behavior of medium manganese steels with different manganese additions. *Materials Science and Engineering: A* **729**, 496-507 (2018).
33. Garcia, C. I. & DeArdo, A. J. Formation of austenite in 1.5 pct Mn steels. *Metallurgical Transactions A* **12**, 521-530 (1981).
34. Hellman, O. C., Vandenbroucke, J. A., Rüsing, J., Isheim, D. & Seidman, D. N. Analysis of three-dimensional atom-probe data by the proximity histogram. *Microscopy and Microanalysis* **6**, 437-444 (2000).
35. Chen, Y.-S. *et al.* Direct observation of individual hydrogen atoms at trapping sites in a ferritic steel. *Science* **355**, 1196-1199 (2017).
36. Breen, A. J. *et al.* Solute hydrogen and deuterium observed at the near atomic scale in high-strength steel. *Acta Materialia* **188**, 108-120 (2020).
37. Chang, Y. *et al.* Ti and its alloys as examples of cryogenic focused ion beam milling of environmentally-sensitive materials. *Nature Communications* **10**, 942 (2019).

38. Yan, F. *et al.* Atomic-scale investigation of hydrogen distribution in a TiMo alloy. *Scripta Materialia* **162**, 321-325 (2019).
39. Kingham, D. R. The post-ionization of field evaporated ions: a theoretical explanation of multiple charge states. *Surface Science* **116**, 273-301 (1982).
40. Gault, B., Moody, M. P., Cairney, J. M. & Ringer, S. P. *Atom Probe Microscopy* (Springer, New York, 2012).
41. Chang, Y. *et al.* Characterizing solute hydrogen and hydrides in pure and alloyed titanium at the atomic scale. *Acta Materialia* **150**, 273-280 (2018).
42. Ryu, J. H., Chun, Y. S., Lee, C. S., Bhadeshia, H. & Suh, D. W. Effect of deformation on hydrogen trapping and effusion in TRIP-assisted steel. *Acta Materialia* **60**, 4085-4092 (2012).
43. Perng, T., Johnson, M. & Altstetter, C. Influence of plastic deformation on hydrogen diffusion and permeation in stainless steels. *Acta Metallurgica* **37**, 3393-3397 (1989).
44. Koyama, M., Tasan, C. C., Akiyama, E., Tsuzaki, K. & Raabe, D. Hydrogen-assisted decohesion and localized plasticity in dual-phase steel. *Acta Materialia* **70**, 174-187 (2014).
45. Owczarek, E. & Zakroczymski, T. Hydrogen transport in a duplex stainless steel. *Acta Materialia* **48**, 3059-3070 (2000).
46. Venezuela, J. *et al.* Determination of the equivalent hydrogen fugacity during electrochemical charging of 3.5 NiCrMoV steel. *Corrosion Science* **132**, 90-106 (2018).
47. Sun, B., Krieger, W., Rohwerder, M., Ponge, D. & Raabe, D. Dependence of hydrogen embrittlement mechanisms on microstructure-driven hydrogen distribution in medium Mn steels. *Acta Materialia* **183**, 313-328 (2020).
48. SEP 1970: Test of the Resistance of Advanced High Strength Steels (AHSS) for Automotive Applications Against Production Related Hydrogen Induced Brittle Fracture, 2011.
49. VDA 238-202: Testing of the Susceptibility of Advanced High Strength Steel Sheets to Hydrogen-induced Cracking, 2020.
50. Cornette, D. *et al.* No detrimental impact of car manufacturing process and simulation of vehicle in-service conditions on DP1180 hydrogen embrittlement. *Proceedings of the Steely Hydrogen Conference.* 46-56 (2014).
51. Georges, C. *et al.* Development of electro galvanized AHSS with tensile strength of 1200 MPa for automotive application with no risk of delayed fracture. *Proceedings of the Steely Hydrogen Conference.* 536-541 (2014).
52. Zhu, X., Li, W., Zhao, H., Wang, L. & Jin, X. Hydrogen trapping sites and hydrogen-induced cracking in high strength quenching & partitioning (Q&P) treated steel. *International Journal of Hydrogen Energy* **39**, 13031-13040 (2014).
53. Ronevich, J. A., Speer, J. G. & Matlock, D. K. Hydrogen embrittlement of commercially produced advanced high strength sheet steels. *SAE International Journal of Materials and Manufacturing* **3**, 255-267 (2010).
54. Bergmann, C. *et al.* Hydrogen embrittlement resistance evaluation of advanced high strength steels in automotive applications. *Proceedings of the Third International Conference on Metals and Hydrogen*; *Steelyhydrogen 2018*, 2018.
55. Kim, H.-J. *et al.* Diffusible hydrogen behavior and delayed fracture of cold rolled martensitic steel in consideration of automotive manufacturing process and vehicle service environment. *Journal of Materials Research and Technology* **9**, 13483-13501 (2020).
56. Uno, N. et al., *Super High Strength Bolt "SHTB®"*, Nippon Steel Technical Report, No. 97, 95-104 (2008).
57. Launey, M. E. & Ritchie, R. O. On the fracture toughness of advanced materials. *Advanced Materials* **21**, 2103-2110 (2009).

58. Tekkaya, A., Bouchard, P.-O., Bruschi, S. & Tasan, C. Damage in metal forming. *CIRP Annals* **69**, 600-623 (2020).
